# Hepatocyte‐Derived FGF1 Alleviates Isoniazid and Rifampicin‐Induced Liver Injury by Regulating HNF4*α*‐Mediated Bile Acids Synthesis

**DOI:** 10.1002/advs.202408688

**Published:** 2024-12-27

**Authors:** Qian Lin, Jiaren Zhang, Jie Qi, Jialin Tong, Shenghuan Chen, Sudan Zhang, Xingru Liu, Huatong Lou, Jiaxuan Lv, Ruoyu Lin, Junjun Xie, Yi Jin, Yang Wang, Lei Ying, Jiamin Wu, Jianlou Niu

**Affiliations:** ^1^ School of Pharmaceutical Sciences Wenzhou Medical University Wenzhou Zhejiang 325035 China; ^2^ Department of Pharmacy Sir Run Run Shaw Hospital School of Medicine Zhejiang University Hangzhou Zhejiang 310016 China; ^3^ Department of Pathology The First Affiliated Hospital of Wenzhou Medical University Wenzhou Zhejiang 325035 China; ^4^ School of Basic Medical Sciences Wenzhou Medical University Wenzhou Zhejiang 325035 China

**Keywords:** anti‐tuberculosis drug‐induced liver injury, bile acids biosynthesis, FGFR4, Fibroblast growth factors, HNF4*α*

## Abstract

Isoniazid and rifampicin co‐therapy are the main causes of anti‐tuberculosis drug‐induced liver injury (ATB‐DILI) and acute liver failure, seriously threatening human health. However, its pathophysiology is not fully elucidated. Growing evidences have shown that fibroblast growth factors (FGFs) play a critical role in diverse aspects of liver pathophysiology. The aim of this study is to investigate the role of FGFs in the pathogenesis of isoniazid (INH) and rifampicin (RIF)‐induced liver injury. Through systematic screening, this study finds that hepatic FGF1 expression is significantly downregulated in both mouse model and human patients challenged with INH and RIF. Hepatocyte‐specific *Fgf1* deficiency exacerbates INH and RIF‐induced liver injury resulted from elevated bile acids (BAs) synthases and aberrant BAs accumulation. Conversely, pharmacological administration of the non‐mitogenic FGF1 analog – FGF1^ΔHBS^ significantly alleviated INH and RIF‐induced liver injury via restoring BAs homeostasis. Mechanically, FGF1 repressed hepatocyte nuclear factor 4*α* (*Hnf4α*) transcription via activating FGF receptor 4 (FGFR4)‐ERK1/2 signaling pathway, thus reducing BAs synthase. The findings demonstrate hepatic FGF1 functions as a negative regulator of BAs biosynthesis to protect against INH and RIF‐induced liver injury via normalizing hepatic BAs homeostasis, providing novel mechanistic insights into the pathogenesis of ATB‐DILI and potential therapeutic strategies for treatment of ATB‐DILI.

## Graphical abstract

This study demonstrates that the significant reduction of hepatic FGF1 in response to isoniazid(INH) and rifampicin(RIF) leads to aberrant accumulation of hepatic bile acids (BAs), ultimately causing drug‐induced liver injury, by inducing FGFR4‐ERK1/2 mediated hepatocyte nuclear factor 4*α* (HNF4*α*) expression, which triggers the transcription of key enzymes for BAs biosynthesis.

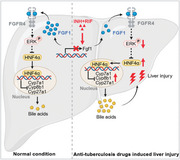

## 1 Introduction

The high incidence of tuberculosis worldwide makes anti‐tuberculosis drug‐induced liver injury (ATB‐DILI) as one of the most common drug‐induced liver injuries.^[^ ^1^, ^2^ ^]^ The first‐line anti‐tuberculosis drugs, including isoniazid and rifampicin, cause severe liver damage, with a risk of hepatotoxicity ranging from 2% to 28% depending on the populations studied, treatment regimens used, and testing strategies.^[^ ^3^ ^]^ Internationally recognized criteria for the definition of ATB‐DILI is when the patient's serum aminotransferase level is 3–5 times higher than the upper limit of normal after taking anti‐tuberculosis drugs.^[^ ^4^ ^]^ The occurrence of severe hepatotoxicity may interrupt or cease the anti‐tuberculosis treatment, reduce its effectiveness, and in severe cases, lead to liver failure and even patient death.^[^ ^5^, ^6^ ^]^

Despite decades of exploration and research, the molecular mechanism of isoniazid and rifampicin‐induced hepatotoxicity has not yet been fully elucidated. Most studies attribute the hepatotoxicity of isoniazid to its metabolites: acetyl hydrazine and hydralazine.^[^ ^3^, ^7^, ^8^ ^]^ The main effect of rifampicin may be to promote the hepatotoxicity of isoniazid, probably through donation of an acetyl group to isoniazid thereby accelerating its metabolism to acetyl hydrazine.^[^ ^7^ ^]^ Although pyrazinamide is an essential component of anti‐tuberculosis drugs, preclinical studies have shown that the addition of pyrazinamide to the isoniazid and rifampicin co‐therapy failed to aggravate drug‐induced liver injury in mice.^[^ ^9^, ^10^ ^]^ Therefore, combined oral administration of isoniazid and rifampicin has become a well‐established ATB‐DILI mouse model, which is widely‐used to clarify the pathogenic mechanism and explore potential targets for intervention of ATB‐DILI.^[^ ^11^, ^12^ ^]^ Using this model, mitochondrial oxidative stress^[^ ^13^ ^]^ and protoporphyrin IX (PPIX) accumulation^[^ ^11^ ^]^ were revealed to play critical roles in the pathogenesis of isoniazid and rifampicin‐induced hepatotoxicity. Recently, growing evidence from preclinical mouse experiments and clinical patients suggest that treatment with anti‐tuberculosis drugs, especially isoniazid and rifampicin co‐therapy, causes hepatic bile acids (BAs) accumulation, thus in turn inducing liver injury.^[^ ^10^, ^14^, ^15^ ^]^ However, how isoniazid and rifampicin co‐therapy induces imbalance in hepatic BAs homeostasis and its underlying molecular mechanisms remain unknown.

Eighteen members of mammalian fibroblast growth factors (FGFs), which are grouped into five paracrine subfamilies (FGF1, FGF4, FGF7, FGF8 and FGF9) and one endocrine‐acting subfamily (FGF19), carry out their pleiotropic functions in embryonic development, wound repair, angiogenesis and metabolic regulation.^[^ ^16^, ^17^, ^18^ ^]^ They bind, and induce the dimerization and activation of FGF receptors (FGFRs), which are encoded by four distinct genes (*Fgfr1*‐*Fgfr4*).^[^ ^16^ ^]^ It is well documented that the loss of function of several FGFs plays an essential role in the pathogenesis of various liver diseases, including non‐alcoholic fatty liver disease (NAFLD)/non‐alcoholic steatohepatitis (NASH), alcoholic fatty liver disease, liver ischemia‐reperfusion and cholestatic liver injury, whereas their exogeneous administrations have many beneficial effects on various liver diseases.^[^ ^19^, ^20^, ^21^, ^22^ ^]^ Notably, endocrine FGF21 prevents the onset and progression of NAFLD by inhibiting liver fat accumulation, excessive inflammation, hepatocyte damage and fibrosis, becoming highly promising candidate drugs.^[^ ^20^, ^23^, ^24^ ^]^ Besides, paracrine FGF1 reverses diet‐induced hepatic steatosis by activating the FGFR4‐AMPK*α* signaling axis in the NAFLD mouse model.^[^ ^25^ ^]^ However, the role of FGFs in the pathogenesis of ATB‐DILI remains unknown.

In this study, we found that isoniazid and rifampicin co‐treatment resulted in a significant reduction in hepatic *Fgf1* in both mouse model and human patients. Hepatocyte‐specific knockout of *Fgf1* exacerbates isoniazid and rifampicin‐induced liver injury, probably resulting from elevated BAs synthases and hepatic BAs accumulation. Pharmacological administration of the non‐mitogenic FGF1 analog – FGF1^ΔHBS^ significantly alleviated isoniazid and rifampicin‐induced liver injury via restoring BAs homeostasis. The mechanistic study reveals that FGF1 probably regulated BAs homeostasis by inhibiting FGFR4‐ERK1/2‐HNF4*α* signaling axis‐mediated transcription of BAs synthases. Taken together, our study demonstrates that hepatic paracrine FGF1 serves as a negative regulator of BAs synthesis to maintain BAs homeostasis and protect against isoniazid and rifampicin‐induced liver injury, providing potential therapeutic strategies for the prevention and treatment of ATB‐DILI.

## 2 Results

### 2.1 Hepatic FGF1 Expression was Significantly Reduced in both Mice and Patients Challenged with Isoniazid and Rifampicin

**Figure 1:**
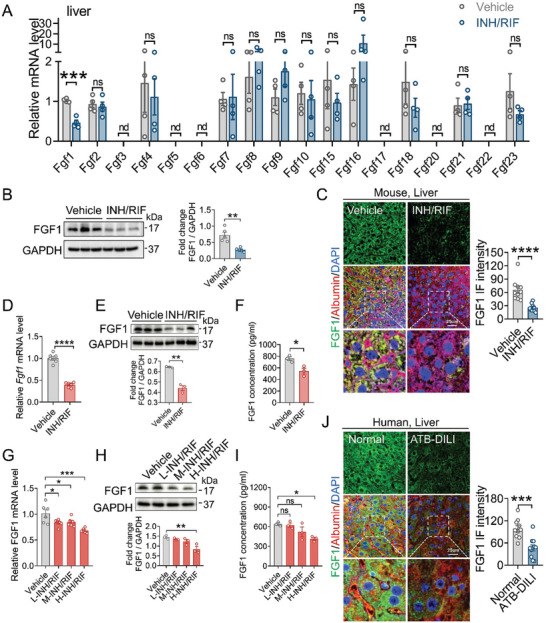
Hepatic FGF1 was down‐regulated by INH and RIF. A–C) Eight‐week‐old male C57BL/6J mice were challenged with 135 mg kg^−1^ isoniazid and 270 mg kg^−1^ rifampicin or vehicle by gavage for three weeks. At the end of this experiment, liver tissues and serum samples were collected for analysis. (A) Relative mRNA levels of all *Fgfs* in the liver tissues from vehicle and INH and RIF‐treated mice were detected by qRT‐PCR and normalized to that of *β‐Actin* (*n* = 4). (B) The hepatic FGF1 expression was determined by western blotting analysis and its semi‐quantitation using Image J. The relative FGF1 expression level was determined after normalization with GAPDH (*n* = 5). (C) Representative image and quantification of liver sections stained with FGF1 (green), albumin (red) and DAPI (blue) from the vehicle and INH and RIF‐treated mice (*n* = 10, two fields per mouse). The FGF1 stains were quantified using Image J software. Scale bar = 25 µm. D–F) The expression level of FGF1 was detected by qRT‐PCR (D) (*n* = 6) and western blotting analysis (E) (*n* = 3) in primary hepatocytes extracted from vehicle and INH/RIF‐treated mice. The concentrations of FGF1 in the cell supernatant were determined by ELISA (F) (*n* = 3). G–I) Primary hepatocytes from C57BL/6J mice were stimulated with DMSO or different dosages of INH and RIF (L‐INH/RIF: 4 µg mL^−1^ INH and 8 µg mL^−1^ RIF, M‐INH/RIF: 20 µg mL^−1^ INH and 40 µg mL^−1^ RIF, H‐INH/RIF: 100 µg mL^−1^ INH and 200 µg mL^−1^ RIF) for 6 h. The expression level of FGF1 was detected by qRT‐PCR (G), western blotting (H); the relative *Fgf1* mRNA level was normalized to that of *β‐Actin* (*n* = 6), the relative FGF1 expression level was determined after normalization with GAPDH (*n* = 3). FGF1 levels in the supernatant were determined by ELISA (I) (*n* = 3). J) The expression of FGF1 (green), hepatocyte marker albumin (red) and DAPI (blue) were detected by IF staining in the liver tissues from healthy individuals and patients with ATB‐DILI (*n* = 10–11). The IF intensity was quantified using Image J software. Scale bar = 25 µm. Data are presented as mean ± SEM; (A, D, F, right panel of B, C, J, lower panel of E) two‐tailed unpaired *t*‐test; (G, I, lower panel of H) ordinary one‐way ANOVA, followed by Dunnett. ^*^ *p* < 0.05; ^**^ *p* < 0.01; ^***^ *p* < 0.001; ^****^ *p* < 0.0001; ns, not significant; nd, not detectable.

To investigate the role of FGFs in the pathogenesis of isoniazid (INH) and rifampicin (RIF)‐induced liver injury, we first analyzed the expression profiles of all hepatic *Fgfs* in normal C57BL/6J mice and found that *Fgf1* was the most predominantly expressed *Fgf* (Figure S1A, Supporting Information). Furthermore, we performed a systematic analysis of the expression of these FGFs comparatively in response to three‐week INH and RIF co‐treatment, the qRT‐PCR data showed that the hepatic expression level of *Fgf1* was specifically down‐regulated (**Figure** **1**A; Figure S1B–D, Supporting Information). Consistently, western blotting analysis and immunofluorescence (IF) staining confirmed the decreased FGF1 levels in the livers of INH and RIF‐treated mice (Figure 1B,C). The double IF staining using FGF1 and albumin antibody showed that FGF1 is mainly expressed in albumin‐positive hepatocytes, whose expression was significantly reduced in mice after INH and RIF co‐treatment (Figure 1C). Notably, the level of hepatic *Fgf1* mRNA was inversely associated with increases in the serum levels of alanine aminotransferase (ALT) and aspartate aminotransferase (AST) (Figure S1E,F, Supporting Information). Besides, qRT‐PCR, western blotting and ELISA analysis of primary hepatocytes extracted from mice treated with three weeks of INH and RIF also showed that paracrine FGF1 was significantly reduced (Figure 1D,F; Figure S1G, Supporting Information). Furthermore, co‐stimulation with INH and RIF dose‐dependently reduced mRNA and protein expressions of FGF1 in primary hepatocytes (Figure 1G–I). In patients, IF staining showed that co‐treatment with INH and RIF significantly reduced hepatic FGF1 levels, which were inversely correlated with the serum levels of ALT and AST (Figure 1J; Figure S1H,I, Supporting Information). However, the levels of intestinal *Fgf15*, a well‐established hormone physically inhibiting hepatic BAs biosynthesis, did not change in response to INH and RIF challenge (Figure S1J–L, Supporting Information). In addition, serum levels of FGF1 and FGF15/19 remained unchanged in both mouse model and human patients with INH and RIF‐induced liver injury (Figure S1M–P, Supporting Information). Furthermore, we found that serum total bile acid (TBA) levels were significantly increased in both mouse model and human patients (Figure S1Q,R, Supporting Information). Taken together, all these data demonstrate that, compared to other FGFs, hepatic FGF1 expression was selectively reduced in both mice and human patients challenged with INH and RIF, suggesting the loss of hepatic FGF1 may be involved in the pathogenesis of INH and RIF‐induced liver injury.

### 2.2 Hepatocyte‐Specific *Fgf1* Deficiency Exacerbates Hepatic BA Accumulation and Liver Injury under INH and RIF Challenge

To investigate the role of FGF1 in the pathogenesis of INH and RIF‐induced liver injury, wild‐type (WT) mice and whole‐body knockout of *Fgf1* (*Fgf1* ^KO^) mice were administrated with vehicle or INH/RIF by gavage for consecutive three weeks. The qRT‐PCR and western blotting analysis verified the complete knockout of *Fgf1* (Figure S2A,B, Supporting Information). We observed that the serum levels of ALT, AST and total bilirubin (TBIL) in vehicle‐treated WT and *Fgf1* ^KO^ mice were similar (Figure S2C–E, Supporting Information). However, compared with WT mice, *Fgf1* ^KO^ mice displayed significantly increased levels of serum ALT, AST and TBIL under the challenge of INH and RIF (Figure S2C–E, Supporting Information), indicating that knockout of *Fgf1* exacerbates INH and RIF‐induced liver injury.

**Figure 2:**
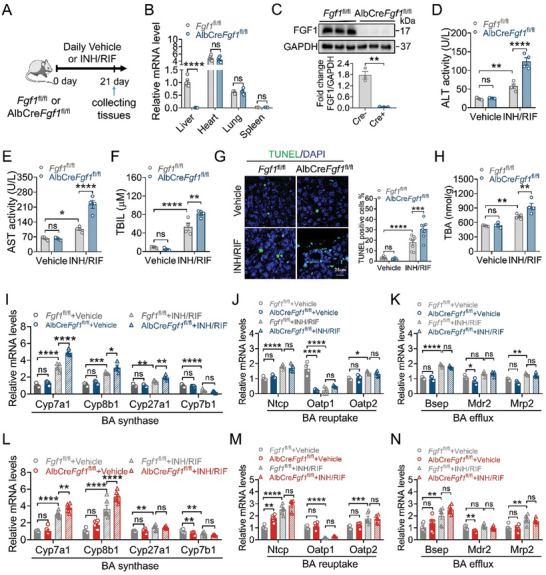
Hepatocyte‐specific *Fgf1* deficiency aggregates liver damage induced by INH and RIF. A) Eight‐week‐old male *Fgf1* ^fl/fl^ and *Fgf1*‐LKO mice were daily challenged with 135 mg kg^−1^ isoniazid and 270 mg kg^−1^ rifampicin or vehicle for three weeks. Liver tissues and serum samples were collected at the end of this experiment. B,C) Liver‐specific *Fgf1* deficiency was verified by qRT‐PCR (B) and western blotting analysis (C). The relative *Fgf1* mRNA level was normalized to that of *β‐Actin* (*n* = 6). FGF1 expression level was determined after normalization with GAPDH (*n* = 3). D–F) Serum ALT (D), AST (E) activity and serum TBIL contents (F) (*n* = 4). G) Representative TUNEL staining of liver sections and quantification by TUNEL positive cells (*n* = 8, two fields per mouse). Scale bar = 25 µm. H) Hepatic TBA contents (*n* = 4). I–K) The mRNA levels of BAs synthase (*Cyp7a1*, *Cyp8b1*, *Cyp27a1* and *Cyp7b1*) (I), BAs transporters of uptake (*Ntcp*, *Oatp1* and *Oatp2*) (J) and efflux (*Bsep*, *Mdr2* and *Mrp2*) (K) in liver tissues were analyzed by qRT‐PCR and normalized to that of *β‐Actin* (*n* = 4). L‐N) The mRNA levels of BAs synthase (*Cyp7a1*, *Cyp8b1*, *Cyp27a1* and *Cyp7b1*) (L), BAs transporters of uptake (*Ntcp*, *Oatp1* and *Oatp2*) (M) and efflux (*Bsep*, *Mdr2* and *Mrp2*) (N) in primary hepatocytes extracted from *Fgf1* ^fl/fl^ and *Fgf1*‐LKO mice treated with vehicle or INH and RIF were analyzed by qRT‐PCR and normalized to that of *β‐Actin* (*n* = 6). Data are presented as mean ± SEM; (D–F, H, I–N, right panel of G) ordinary two‐way ANOVA, followed by Sidak; (B, C) two‐tailed unpaired ^t^‐test. ^*^ *p* < 0.05; ^**^ *p* < 0.01; ^***^ *p* < 0.001; ^****^ *p* < 0.0001; ns, not significant.

To further explore the role of hepatic FGF1 in the pathogenesis of INH and RIF‐induced liver injury, we generated hepatocyte‐specific *Fgf1* deficient mice (AlbCre*Fgf1* ^fl/fl^, termed as *Fgf1*‐LKO) by crossing *Fgf1* ^fl/fl^ and Alb‐Cre mice, followed by INH and RIF challenge for 3 weeks (**Figure** **2**A). The qRT‐PCR, western blotting analysis and double IF staining revealed that FGF1 was specifically and efficiently deleted in the liver of *Fgf1*‐LKO mice (Figure 2B,C; Figure S3A, Supporting Information). Consistent with results from *Fgf1* ^KO^ mice, serum levels of ALT, AST and TBIL were more significantly elevated in *Fgf1*‐LKO mice than *Fgf1* ^fl/fl^ mice after INH and RIF co‐treatment (Figure 2D–F). In line with severe liver damage, ablation of hepatic *Fgf1* substantially aggravated hepatocyte cell death in the liver, as revealed by TUNEL, cleaved caspase 3 IF staining and H&E staining (Figure 2G; Figure S3B,C, Supporting Information).

To decipher the molecular basis by which hepatic *Fgf1* deficiency exacerbates INH and RIF‐induced liver injury, we next analyzed the levels of bile acids (BAs), pro‐inflammatory cytokines and oxidative stress in the livers, which may trigger hepatocyte damage and apoptosis.^[^ ^7^, ^26^, ^27^ ^]^ We found that hepatic *Fgf1* deficiency significantly exacerbated INH and RIF‐induced BAs accumulation in the liver, as determined by hepatic total BAs (TBA) (Figure 2H), without affecting inflammation (Figure S3D,E, Supporting Information) or oxidative stress (Figure S3F–H, Supporting Information). Further analyses of the hepatic expression levels of key enzymes involved in BAs biosynthesis (classical and alternative pathways), transporters of BAs reuptake and efflux showed that under INH and RIF challenge, hepatic *Fgf1* deletion exacerbated elevations of key BAs synthetases, including cholesterol 7a hydroxylase (*Cyp7a1*), sterol 12a hydroxylase (*Cyp8b1*) and sterol 27 hydroxylase (*Cyp27a1*), without affecting levels of BAs reuptake transporters (*Ntcp*, *Oatp1*, *Oatp2*) or efflux transporters (*Bsep*, *Mdr2*, *Mrp2*) (Figure 2I–K). Consistent with the in vivo data, results from primary hepatocytes isolated from INH and RIF‐treated *Fgf1* ^fl/fl^ and *Fgf1*‐LKO mice showed that *Fgf1* deletion significantly increased the levels of key BAs synthetases (Figure 2L), without altering the levels of BAs reuptake or efflux transporters (Figure 2M,N), inflammation (Figure S3I, Supporting Information) or oxidative stress (Figure S3J,K, Supporting Information). Taken together, all these data demonstrate that hepatocyte‐specific *Fgf1* deficiency exacerbates INH and RIF‐induced BAs accumulation and liver injury, probably via upregulating BAs synthesis, indicating that hepatic endogenous FGF1 may act as a negative regulator of BAs synthesis to protect against INH and RIF‐induced liver injury.

### 2.3 Administration of FGF1^ΔHBS^ Alleviates INH and RIF‐Induced Liver Injury via Restoring Hepatic BAs Homeostasis

**Figure 3:**
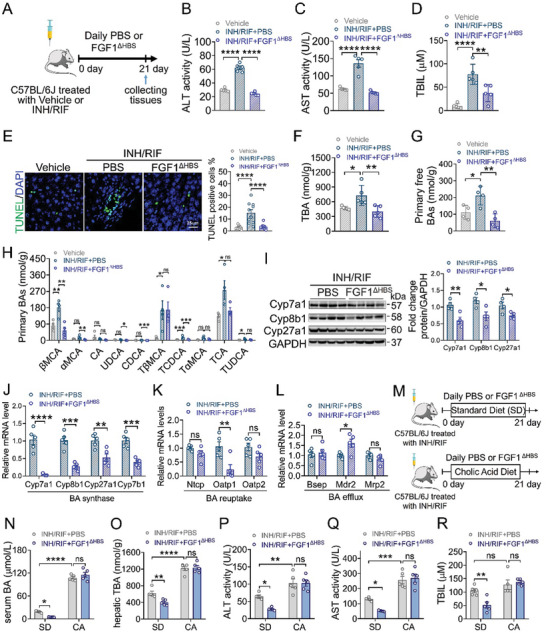
Administration of the non‐mitogenic FGF1 (FGF1^ΔHBS^) alleviated INH and RIF‐induced liver injury by normalizing hepatic BAs homeostasis. A) Eight‐week‐old male C57BL/6J mice were challenged with vehicle or 135 mg kg^−1^ isoniazid and 270 mg kg^−1^ rifampicin by gavage, followed by daily i.p. administration of PBS or FGF1^ΔHBS^ (0.1 mg kg^−1^ BW/day) for three weeks. B‐D) Serum ALT (B), AST (C) and serum TBIL contents (D) (*n* = 5). E) TUNEL staining of liver sections and quantification by TUNEL positive cells (*n* = 10, two fields per mice). Scale bar = 25 µm. F‐H) Hepatic TBA contents (F) (*n* = 5), primary free BAs (G), and detailed components of primary BAs analyzed by mass spectrometry (H) (*n* = 4). I) Hepatic protein expressions of Cyp7a1, Cyp8b1 and Cyp27a1 in the liver tissues from INH and RIF‐treated mice treated with PBS or FGF1^ΔHBS^ were determined by western blotting analysis and its semi‐quantitation using Image J. The relative protein levels were determined after normalization with GAPDH (*n* = 4). J‐L) The mRNA levels of BA synthases (*Cyp7a1*, *Cyp8b1*, *Cyp27a1* and *Cyp7b1*) (J), transporters of reuptake (K) and efflux (L) in the liver tissues from INH and RIF‐treated mice treated with PBS or FGF1^ΔHBS^ were analyzed by qRT‐PCR and normalized to *β‐Actin* (*n* = 5). M) Eight‐week‐old male C57BL/6J mice fed by standard diet (SD) or diet containing 0.5% CA were challenged with vehicle or 135 mg kg^−1^ isoniazid and 270 mg kg^−1^ rifampicin by gavage, followed by daily i.p. administration of PBS or FGF1^ΔHBS^ (0.1 mg kg^−1^ BW/day) for three weeks. N‐R) Serum BA level (N), hepatic TBA contents (O), serum ALT (P), AST (Q) and serum TBIL contents (R) (*n* = 5). Data are presented as mean ± SEM; (B‐D, right panel of E, F‐H) ordinary one‐way ANOVA, followed by Dunnett; (right panel of I, J‐L) two‐tailed unpaired ^t^‐test; (N‐R) ordinary two‐way ANOVA, followed by Sidak. ^*^ *p* < 0.05; ^**^ *p* < 0.01; ^***^ *p* < 0.001; ^****^ *p* < 0.0001; ns, not significant.

Then, we investigated whether exogenous supplementation of recombinant FGF1 could correct hepatic BAs homeostasis and thus alleviate INH and RIF‐induced liver injury. With the consideration that the potent proliferation of wild‐type FGF1 may induce safety risks and hamper the evaluation of the therapeutic effect of FGF1 on INH and RIF‐induced liver injury during chronic administration, we used an engineered non‐mitogenic FGF1 analog (named FGF1^ΔHBS^) reported in our previous study^[^ ^28^ ^]^ in the subsequent experiments. C57BL/6J mice challenged with INH and RIF were daily i.p. injected with FGF1^ΔHBS^ (0.1 mg kg^−1^) or PBS for 3 weeks (**Figure** **3**A). We found that FGF1^ΔHBS^ administration remarkably protected mice against INH and RIF‐induced liver injury, as evidenced by a marked attenuation in the elevations of serum ALT, AST and TBIL levels (Figure 3B–D). Besides, TUNEL staining showed that FGF1^ΔHBS^ administration significantly inhibited the hepatocyte cell death (Figure 3E). Furthermore, FGF1^ΔHBS^ did not induce hepatic cell proliferation as revealed by PCNA and Ki‐67 expressions (Figure S4A,B, Supporting Information).

More importantly, we observed that FGF1^ΔHBS^ administration significantly decreased hepatic TBA content in the INH and RIF‐induced liver injury mouse model (Figure 3F). Detailed analysis of hepatic BA compositions by targeted mass spectrometry showed treatment of INH and RIF significantly elevated the levels of hydrophobic primary BAs, especially *β*‐muricholic acid (*β*MCA) (the main primary component of BAs in mice), tauro‐*β*MCA (T*β*MCA) and tauro‐CA (TCA); whereas FGF1^ΔHBS^ treatment markedly reduced the levels of hepatic primary BAs even to the normal ranges, including *β*MCA, *α*MCA, UDCA, CDCA, tauro‐CA (TCA) and tauro‐CDCA (TCDCA) (Figure 3G,H). Further, qRT‐PCR and western blotting analyses showed that FGF1^ΔHBS^ significantly reduced the levels of key BAs synthetases without affecting BAs reuptake or efflux transporters (Figure 3I–L). Consistently, in primary hepatocytes isolated from INH and RIF‐treated mice and HepG2 cells challenged with INH and RIF, FGF1^ΔHBS^ stimulation remarkably inhibited the levels of key BAs synthetases, without affecting the levels of BAs reuptake or efflux transporters (Figure S5, Supporting Information). Taken together, all these data suggest that FGF1^ΔHBS^ alleviates INH and RIF‐induced liver injury mainly via restoring hepatic BAs homeostasis.

To further support this notion, we performed a cholic acid (CA) diet experiment in INH and RIF‐induced liver injury mouse model (Figure 3M). The results showed that FGF1^ΔHBS^ treatment significantly decreased serum and hepatic TBA contents in the standard diet (SD)‐fed mice but not CA diet‐fed mice (Figure 3N,O), indicating CA diet remarkably increased basal levels of serum and hepatic TBA to forcibly compromise the effect of FGF1^ΔHBS^ on maintaining BAs homeostasis. As expected, administration of FGF1^ΔHBS^ alleviated INH and RIF‐induced liver injury in the standard diet (SD)‐fed mice but not CA diet‐fed mice (Figure 3P–R), further demonstrating that the protective effect of FGF1^ΔHBS^ on INH and RIF‐induced liver injury is probably attributed to the restoration of hepatic BAs homeostasis.

### 2.4 HNF4*α* Mediated the BAs Regulatory Activity and Protective Effects of FGF1^ΔHBS^ in the INH and RIF‐Induced Liver Injury Mouse Model

**Figure 4:**
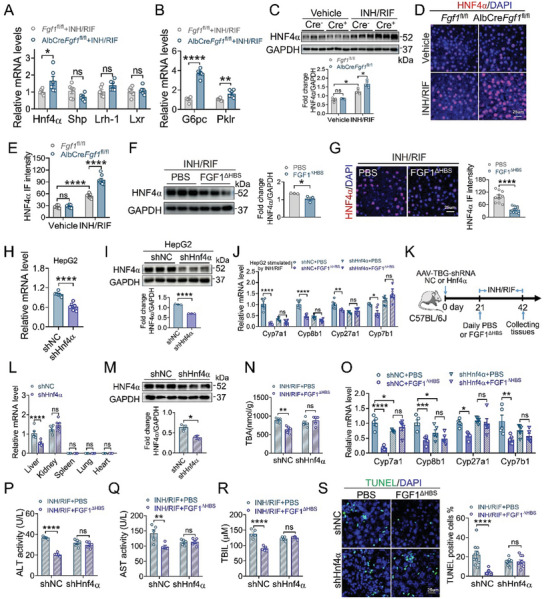
HNF4*α* mediated the BAs regulatory activity of FGF1 in the INH and RIF‐induced liver injury mouse model. A,B) The mRNA levels of *Hnf4α*, *Shp*, *Lrh‐1*, *Lxr* (A) and the target genes of *Hnf4α* (*G6pc* and *Pklr*) (B) in primary hepatocytes extracted from INH and RIF‐treated *Fgf1* ^fl/fl^ and *Fgf1*‐LKO mice were analyzed by qRT‐PCR and normalized to that of *β‐Actin* (*n* = 6). C‐E) The expression levels of HNF4*α* in the liver tissues from vehicle or INH and RIF‐treated *Fgf1* ^fl/fl^ and *Fgf1*‐LKO mice were determined by western blotting analysis (C) and IF staining (D‐E). The relative HNF4*α* expression level was determined after normalization with GAPDH (*n* = 3). The HNF4*α* stains were quantified using Image J software (E) (*n* = 8, two fields per mice). Scale bar = 25 µm. F,G) The expression levels of HNF4*α* in the liver tissues from ATB‐DILI mice treated with PBS or FGF1^ΔHBS^ were determined by western blotting analysis (F) and IF staining (G). The relative HNF4*α* expression level was determined after normalization with GAPDH (*n* = 3). The HNF4*α* stains were quantified using Image J software (*n* = 10, two fields per mice). Scale bar = 25 µm. H,I) Knockdown of *Hnf4α* in HepG2 cells transfected with shRNA were verified by qRT‐PCR (H) and western blotting analysis (I). The relative *Hnf4α* mRNA level was normalized to that of *β‐Actin* (*n* = 6). The relative HNF4*α* expression level was determined after normalization with GAPDH (*n* = 3). J) The mRNA levels of BAs synthases in shNC or sh*Hnf4α*‐transfected HepG2 cells after treated with PBS or FGF1^ΔHBS^ for 6 h were analyzed by qRT‐PCR and normalized to *β‐Actin* (*n* = 6). K) Male C57BL/6J mice transfected by AAV‐TBG‐shRNA NC (shNC) or AAV‐TBG‐shRNA *Hnf4α* (sh*Hnf4α*) were challenge with INH and RIF, followed by daily i.p. administration of PBS or FGF1^ΔHBS^ for three weeks. Liver tissues and serum were collected at the end of this experiment. L,M) Liver‐specific *Hnf4α* knockdown were verified by qRT‐PCR (L) and western blotting analysis (M). The relative *Hnf4α* mRNA level was normalized to that of *β‐Actin* (*n* = 6). The relative HNF4*α* expression level was determined after normalization with GAPDH (*n* = 3). N) Hepatic TBA contents (*n* = 5). O) Hepatic mRNA levels of *Cyp7a1*, *Cyp8b1*, *Cyp27a1* and *Cyp7b1* were determined by qRT‐PCR and normalized to *β‐Actin* (*n* = 5). P‐R) Serum ALT (P), AST (Q), serum TBIL contents (R) (*n* = 5). S) TUNEL staining of liver sections and quantification by TUNEL positive cells (*n* = 10, two fields per mice). Scale bar = 25 µm. Data are presented as mean ± SEM; (A, B, right panel of F, G, H, lower panel of I, L, M) two‐tailed unpaired ^t^‐test; (lower panel of C, E, J, N‐R, right panel of S) ordinary two‐way ANOVA, followed by Sidak. ^*^ *p* < 0.05; ^**^ *p* < 0.01; ^***^ *p* < 0.001; ^****^ *p* < 0.0001; ns, not significant.

To explore how hepatic FGF1 regulates the transcriptional levels of BA synthases, primary hepatocytes were isolated from *Fgf1* ^fl/fl^ and *Fgf1*‐LKO mice after three week co‐treatment of INH and RIF to analyze the levels of several transcription factors, including small heterodimer partner (SHP), liver X receptor (LXR), liver receptor homolog‐1 (LRH‐1) and hepatocyte nuclear factor 4*α* (HNF4*α*), which regulate the expressions of enzymes involved in BAs biosynthesis.^[^ ^29^, ^30^, ^31^ ^]^ We observed that *Fgf1* deficiency specifically and markedly increased the levels of *Hnf4α* and its target genes including glucose‐6‐phosphatase (*G6pc*) and pyruvate kinase L/R (*Pklr*), without affecting *Shp*, *Lrh‐1* or *Lxr* (**Figure** **4**A,B; Figure S6A, Supporting Information). In contrast, FGF1^ΔHBS^ treatment selectively decreased HNF4*α* level in primary hepatocytes derived from INH and RIF‐treated mice (Figure S6B–D, Supporting Information) and HepG2 cells challenged with INH and RIF (Figure S6E,F, Supporting Information). In line with these in vitro data, hepatocyte‐specific *Fgf1* deficiency significantly increased hepatic HNF4*α* levels in INH and RIF‐treated mice (Figure 4C–E), whereas FGF1^ΔHBS^ treatment decreased HNF4*α* expression in the livers (Figure 4F,G). Taken together, all these results indicate that hepatic FGF1 acts as an upstream suppressor of HNF4*α* under the challenge of INH and RIF.

We next explored whether the HNF4*α* mediated the BAs regulatory activity of FGF1. Consistent with previous studies,^[^ ^32^, ^33^ ^]^ silencing of *Hnf4α* significantly reduced the basal levels of *Cyp7a1*, *Cyp8b1* and *Cyp27a1* in HepG2 cells (Figure 4H–J). More importantly, silencing of *Hnf4α* compromised the inhibitory effects of FGF1^ΔHBS^ on BA syntheses (Figure 4J). To further confirm this in vivo, C57BL/6J mice transfected with recombinant AAV8‐TBG‐shRNA *Hnf4α* (sh*Hnf4α*) or AAV‐TBG‐shRNA negative control (shNC) were challenged with INH and RIF, followed by daily i.p. administration of FGF1^ΔHBS^ or PBS for three weeks (Figure 4K). Liver‐specific knockdown of *Hnf4α* was verified by qRT‐PCR and western blotting analysis (Figure 4L,M). Consistent with cell‐based data, the levels of hepatic total bile acids (TBA) and BAs synthases (*Cyp7a1*, *Cyp8b1*, *Cyp27a1* and *Cyp7b1*) in the shNC group were significantly reduced after FGF1^ΔHBS^ treatment. However, FGF1^ΔHBS^ failed to inhibit BAs synthases or correct hepatic BAs homeostasis in sh*Hnf4α*‐transfected mice (Figure 4N,O), demonstrating that hepatic HNF4*α* is required for the inhibitory effect of FGF1^ΔHBS^ on BAs biosynthesis. In addition, we noticed that compared with that of the shNC group, the basal levels of BAs synthases and liver injury tended to be reduced in sh*Hnf4α*‐transfected mice, but there was no significant difference, which is likely due to the inefficiency of *Hnf4α* knockdown or other compensatory effects in liver tissue. Furthermore, *Hnf4α* knockdown abolished the hepatoprotective effects of FGF1^ΔHBS^, as determined by the levels of serum ALT, AST and TBIL (Figure 4P–R). TUNEL staining of liver tissues further confirmed that FGF1^ΔHBS^ failed to alleviate hepatocyte cell death in sh*Hnf4α*‐transfected mice (Figure 4S). Taken together, these results support a requirement for HNF4*α*‐mediated BAs biosynthesis in the protective effect of FGF1^ΔHBS^ in INH and RIF‐induced liver injury.

### 2.5 FGF1^ΔHBS^ Inhibits *Hnf4α* Transcription via Activating FGFR4‐ERK1/2 Signaling Axis in Hepatocyte

**Figure 5:**
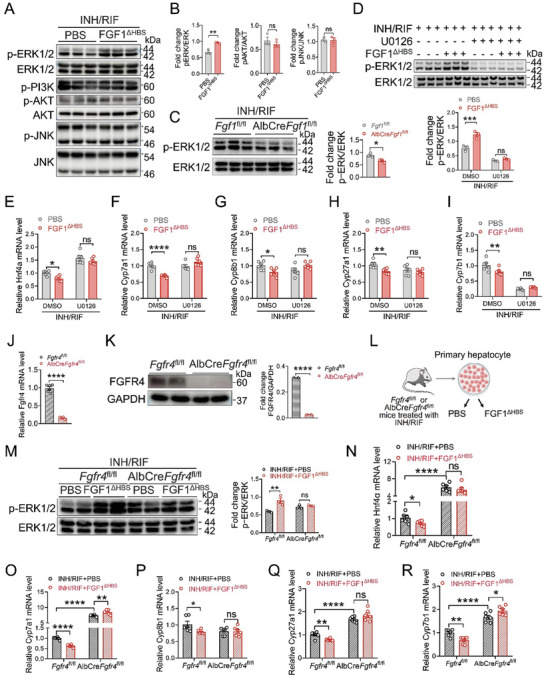
FGF1^ΔHBS^ inhibited HNF4*α*‐mediated BA biosynthesis via FGFR4‐EKR1/2 pathway. A,B) Primary hepatocytes extracted from INH and RIF‐treated mice were stimulated by PBS or 1000 ng mL^−1^ FGF1^ΔHBS^ for 5 min. The protein expression of p‐ERK1/2, ERK1/2, p‐PI3K, p‐AKT, AKT, p‐JNK and JNK were determined by western blotting analysis and its semi‐quantitation using Image J. The relative protein levels were determined after normalization with corresponding total protein (*n* = 3). C) The activation of ERK1/2 in INH and RIF‐treated *Fgf1* ^fl/fl^ and *Fgf1* ^fl/fl^‐LKO mice were detected by western blotting analysis and its semi‐quantitation using Image J. The relative p‐ERK1/2 protein levels were determined after normalization with ERK (*n* = 3). D,E) Primary hepatocytes extracted from INH and RIF‐treated mice were pretreated with 10 uM U0126 for 2 h, followed by PBS or 1000 ng mL^−1^ FGF1^ΔHBS^ stimulation for 6 h. The protein levels of p‐ERK1/2 and ERK1/2 were determined by western blotting analysis (D) and its semi‐quantitation by Image J (*n* = 3). The mRNA levels of *Hnf4α* were analyzed by qRT‐PCR (E) and normalized to *β‐Actin* (*n* = 6). F‐I) The mRNA levels of *Cyp7a1* (F), *Cyp8b1* (G), *Cyp27a1* (H) and *Cyp7b1* (I) in primary hepatocytes were analyzed by qRT‐PCR and normalized to *β‐Actin* (*n* = 6). J,K) Deficiency of *Fgfr4* was verified by qRT‐PCR (J) and western blotting analysis (K). The relative *Fgfr4* mRNA level was normalized to that of *β‐Actin* (*n* = 6). The relative FGFR4 expression level was determined after normalization with GAPDH (*n* = 2). L) Primary hepatocytes extracted from INH and RIF‐treated *Fgfr4* ^fl/fl^ or *Fgfr4*‐LKO mice were stimulated by PBS or 1000 ng mL^−1^ FGF1^ΔHBS^. M) The protein levels of p‐ERK1/2 and ERK1/2 in primary hepatocytes were determined by western blotting analysis and its semi‐quantitation using Image J. The relative p‐ERK1/2 expression level was determined after normalization with ERK1/2 (*n* = 3). N) The mRNA levels of *Hnf4α* in primary hepatocytes were analyzed by qRT‐PCR and normalized to *β‐Actin* (*n* = 6). O‐R) The mRNA levels of *Cyp7a1* (O), *Cyp8b1* (P), *Cyp27a1* (Q) and *Cyp7b1* (R) in primary hepatocytes were analyzed by qRT‐PCR and normalized to *β‐Actin* (*n* = 6). Data are presented as mean ± SEM; (B, right panel of C and L, K) two‐tailed unpaired ^t^‐test; (E‐I, lower panel of D, right panel of M, N‐R) ordinary two‐way ANOVA, followed by Sidak. ^*^ *p* < 0.05; ^**^ *p* < 0.01; ^***^ *p* < 0.001; ^****^ *p* < 0.0001; ns, not significant.

Depending on cell types in distinct tissues, the classical FGFR downstream signaling pathways activated by FGF1 include Ras/Raf‐MEK‐MAPKs (mitogen‐activated protein kinases) pathway and phosphatidylinositol‐3 kinase/protein kinase B (PI3K/AKT) pathway.^[^ ^17^, ^34^ ^]^ Therefore, we explored which signaling pathway was activated by FGF1^ΔHBS^ to reduce transcription level of *Hnf4α* in the liver and found that FGF1^ΔHBS^ only activated ERK1/2 without affecting PI3K, AKT, and JNK signaling in HepG2 cells challenged with INH and RIF (Figure S7A, Supporting Information). Consistently, blocking of ERK1/2 signaling pathway by its inhibitor significantly compromised the inhibitory effect of FGF1^ΔHBS^ on *Hnf4α* transcription (Figure S7B,C, Supporting Information). To further confirm this, we further analyzed these signaling pathways in primary hepatocytes (isolated from INH and RIF‐treated mice) treated with FGF1^ΔHBS^ or PBS. We found that FGF1^ΔHBS^ specifically activates ERK1/2 signaling pathway, without affecting the expressions of p‐JNK, p‐PI3K or p‐AKT in primary hepatocytes (**Figure** **5**A,B). In contrast, hepatocyte‐specific *Fgf1* deficiency significantly reduced hepatic ERK1/2 phosphorylation in INH and RIF‐treated mice compared to *Fgf1* ^fl/fl^ mice (Figure 5C). Notably, the ability of FGF1^ΔHBS^ to suppress transcriptions of *Hnf4α* and BAs synthases were all compromised by ERK1/2 inhibitor U016 (Figure 5D–I). In addition, we found that ERK1/2 inhibition did not increase basal levels of *Cyp7a1* and *Cyp8b1*, which was probably due to the raised threshold of BA synthetases induced by INH and RIF. Taken together, all these results strongly suggest that FGF1^ΔHBS^ inhibits *Hnf4α* transcriptions via activating ERK1/2 pathway.

Given that FGFR4 is the most expressed FGFR of four major subtypes of FGFRs in hepatocytes,^[^ ^19^, ^35^ ^]^ we speculate that FGFR4 probably mediates the activation of ERK1/2 by FGF1^ΔHBS^. To test this hypothesis, we generated hepatocyte‐specific *Fgfr4* deficient mice (AlbCre*Fgfr4* ^fl/fl^, termed as *Fgfr4*‐LKO) by crossing *Fgfr4* ^fl/fl^ and Alb‐Cre mice and validated knockout of *Fgfr4* in hepatocytes by qRT‐PCR and western blotting analyses (Figure 5J,K). Primary hepatocytes isolated from *Fgfr4* ^fl/fl^ or *Fgfr4*‐LKO mice (under the challenge of INH and RIF for three weeks) were treated with FGF1^ΔHBS^ or PBS (Figure 5L). We found that depletion of hepatic *Fgfr4* abrogated FGF1^ΔHBS^‐activated ERK1/2 phosphorylation (Figure 5M) and led to significant increases in basal mRNA levels of *Hnf4α* and BAs synthases (Figure 5N–R). Consequently, FGF1^ΔHBS^ failed to decrease mRNA levels of *Hnf4α* and BAs synthases in *Fgfr4‐*LKO mice (Figure 5N–R). Taken together, all these data demonstrate that FGF1^ΔHBS^ reduced the transcription of *Hnf4α* via FGFR4‐ERK1/2 signaling axis in hepatocytes.

### 2.6 Hepatic *Fgfr4* Deficiency Abolished the Protective Effects of FGF1^ΔHBS^ on INH and RIF‐Induced Liver Injury

**Figure 6:**
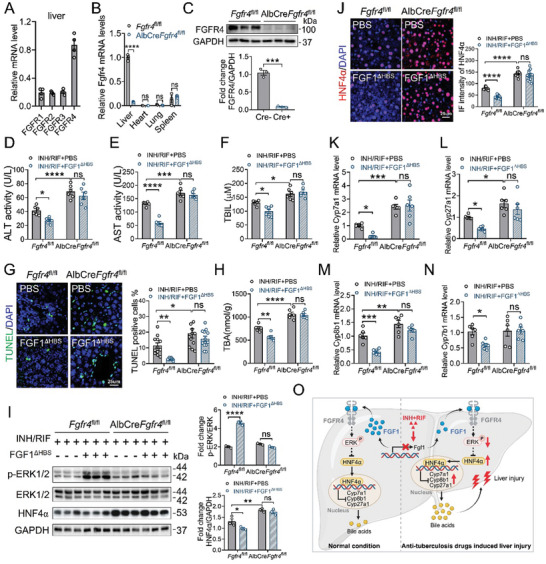
Hepatic Fgfr4 deficiency abolished the protective effects of FGF1^ΔHBS^ in the mouse model of INH and RIF‐induced liver injury. A) The expression profile of all FGFRs in the liver tissues of normal C57BL/6J mice by qRT‐PCR analysis. The relative *Fgfr4* mRNA level was normalized to that of *β‐Actin* (*n* = 4). B,C) Liver‐specific *Fgfr4* deficiency was verified by qRT‐PCR (B) and western blotting analysis (C). The relative *Fgfr4* mRNA level was normalized to that of *β‐Actin* (*n* = 3). D‐N) Eight‐week‐old male *Fgfr4* ^fl/fl^ and *Fgfr4*‐LKO mice were challenge with 135 mg kg^−1^ isoniazid and 270 mg kg^−1^ rifampicin by gavage, followed by i.p. daily administration of PBS or FGF1^ΔHBS^ (0.1 mg kg^−1^ BW/day) for three weeks (*n* = 6). Liver tissues and serum were collected for analysis at the end of this experiment. (D‐F) Serum ALT (D), AST (E), serum TBIL contents (F). (G) TUNEL staining of liver sections and quantification by TUNEL positive cells (*n* = 12, two fields per mice). Scale bar = 25 µm. (H) Hepatic TBA contents. (I) The protein levels of p‐ERK1/2, ERK1/2 and HNF4*α* in liver tissues were determined by western blotting analysis and its semi‐quantitation using Image J (*n* = 3). (J) Analysis of the expression levels of HNF4*α* in liver tissues from the *Fgfr4* ^fl/fl^ and *Fgfr4*‐LKO mice by IF staining. The IF intensity was quantified using ImageJ software (*n* = 12, two fields per mice). Scale bar = 25 µm. (K‐N) The mRNA levels of Cy*p7a1* (K), *Cyp8b1* (L), *Cyp27a1* (M) and *Cyp7b1* (N) in liver tissues were analyzed by qRT‐PCR and normalized to *β‐Actin* (*n* = 6). O) A graphical model depicting the roles of FGF1 in protecting against ATB‐DILI by maintaining FGFR4‐ERK1/2‐HNF4*α* signaling axis‐mediated BAs homeostasis. Image created using BioRender. Data are presented as mean ± SEM; (D‐F, right panel of G and I and J, H, K‐N) ordinary two‐way ANOVA, followed by Sidak; (B, lower panel of C) two‐tailed unpaired ^t^‐test. ^*^ *p* < 0.05; ^**^ *p* < 0.01; ^***^ *p* < 0.001; ^****^ *p* < 0.0001, ns, not significant.

Consistent with previous studies,^[^ ^25^, ^35^ ^]^ *Fgfr4* is the predominant *Fgfrs* in the liver of normal mice (**Figure** **6**A). To further determine whether FGFR4 is essential for the protective effects of FGF1^ΔHBS^ on INH and RIF‐induced liver injury, *Fgfr4* ^fl/fl^ or *Fgfr4*‐LKO mice challenged with INH and RIF were daily i.p. injected with FGF1^ΔHBS^ or PBS for three weeks. Liver specific deletion of *Fgfr4* was identified by qRT‐PCR and western blotting analysis (Figure 6B,C). Compared to *Fgfr4* ^fl/fl^ mice, the deletion of *Fgfr4* in the liver exacerbated INH and RIF‐induced liver injury, which was manifested by increases in serum ALT, AST and TBIL levels. More importantly, the protective effect of FGF1^ΔHBS^ on INH and RIF‐induced liver injury in *Fgfr4* ^fl/fl^ (as revealed by reduced serum ALT, AST and TBIL levels, hepatocyte cell death) was mostly abrogated by *Fgfr4* ^fl/fl^ deficiency (Figure 6D–G). Hepatocyte specific *Fgfr4* deletion resulted in a significant increase in hepatic BAs accumulation, which was not reversed by FGF1^ΔHBS^ treatment (Figure 6H). Furthermore, FGF1^ΔHBS^ failed to activate ERK1/2 phosphorylation and reduce HNF4*α* expression level due to the loss of *Fgfr4* in hepatocytes (Figure 6I,J). Consistently, the mRNA levels of *Cyp7a1*, *Cyp8b1*, and *Cyp27a1* could not be inhibited by FGF1^ΔHBS^ in *Fgfr4*‐LKO mice (Figure 6K–N). In addition, we found that administration of recombinant FGF19 (rFGF19), the human ortholog of FGF15, significantly reduced the hepatic levels of total bile acids (TBA) and key BA synthetases (*Cyp7a1* and *Cyp8b1*) under the challenge of INH and RIF (Figure S8A,B, Supporting Information). However, unlike FGF1, rFGF19 increased mRNA and protein levels of HNF4*α*, suggesting that BA regulatory activity of FGF19 is independent of this transcriptional factor (Figure S8C,D, Supporting Information). Taken together, all these results demonstrate that hepatic FGFR4 is required for activating ERK1/2 phosphorylation and subsequently inhibiting the transcription of *Hnf4α* and BAs synthases, which mediates the protective effects of FGF1^ΔHBS^ in INH and RIF‐induced liver injury.

## 3 Discussion

The FGF family plays an important role in liver metabolism and has emerged as a potential therapeutic for treatment of NAFLD/NASH, alcoholic fatty liver disease, cholestatic liver disease, and other liver diseases.^[^ ^19^, ^20^, ^21^, ^22^ ^]^ However, it remains elusive as to whether the loss functions of FGFs are involved in the pathogenesis of ATB‐DILI. In the study, we found that the expression of hepatic FGF1 was significantly reduced in both mice and patients challenged with INH and RIF (Figure 1; Figure S1, Supporting Information). Hepatocyte‐specific *Fgf1* deficiency exacerbated hepatic BAs accumulation and liver injury under the challenge of INH and RIF (Figure 2). In contrast, the pharmacological administration of recombinant non‐mitogenic FGF1 analogue – FGF1^ΔHBS^ markedly alleviated INH and RIF‐induced liver injury by normalizing hepatic BAs homeostasis (Figure 3). Mechanically, FGF1 repressed hepatocyte nuclear factor 4*α* (HNF4*α*)‐mediated BA synthase expression via activating FGFR4‐ERK1/2 signaling pathway (Figures 4, 5, 6). Our study establishes that hepatic paracrine FGF1 serves as a negative regulator of BA biosynthesis under the challenge of INH and RIF, providing novel and deep mechanistic insights into the pathogenesis of ATB‐DILI and potential therapeutic strategies for the intervention and treatment of this disease.

Normally, BAs are amphiphilic detergents that are important for facilitating the absorption of dietary fats and liposoluble vitamins. However, disrupted BAs homeostasis has been demonstrated to contribute to the occurrence and development of a variety of diseases, including inflammatory bowel disease, cholestatic liver damage, and non‐alcoholic fatty liver disease.^[^ ^36^, ^37^, ^38^, ^39^ ^]^ Recently, growing evidences have suggested that hepatic BAs accumulation is closely related to ATB‐DILI.^[^ ^7^, ^10^, ^27^ ^]^ However, there is still a lack of systematic studies to illustrate the mechanism by which anti‐tuberculosis drugs induce the toxic BAs accumulation in the livers. In the study, mass spectrometry analysis of BAs composition in the liver showed that the level of hepatic total bile acids (TBA), especially hydrophobic primary BAs, was significantly elevated by INH and RIF treatment (Figure 3F–H). More importantly, INH and RIF co‐therapy led to a remarkable reduction in hepatic FGF1 level, which was closely related to the aberrant accumulation of BAs (Figures 1 and 2). In contrast, pharmacological administration of FGF1^ΔHBS^ corrected hepatic BAs homeostasis and alleviated INH and RIF‐induced liver injury (Figure 3; Figure S5, Supporting Information). Taken together, all these data strongly support that hepatic loss of *Fgf1* induced by INH and RIF co‐treatment resulted in BAs accumulation in the liver, contributing to the pathogenesis of ATB‐DILI. In addition, we noticed that *Fgf1* ^KO^ mice exhibited less damage than hepatocyte‐specific *Fgf1* deficient (*Fgf1*‐LKO) mice under INH/RIF challenge (Figure 2; Figure S2, Supporting Information), suggesting that *Fgf1* deficiency in other tissues may lead to hepatic protective effects via unknown mechanisms.

BA‐farnesoid X receptor (FXR) signaling axis‐mediated regulation of intestinal FGF15 in mice and its human orthologue FGF19 plays a central role in the maintaining BA homeostasis in steady‐state conditions.^[^ ^23^, ^40^ ^]^ FGF15/FGF19 is secreted from intestinal epithelia cells, reaches the liver via portal circulation and binds with FGFR4‐beta Klotho (KLB) complex to initiate a signaling cascade including c‐Jun amino‐terminal‐kinase, inhibiting the expression of Cyp7a1, the rate‐limiting enzyme for BA biosynthesis.^[^ ^41^ ^]^ It has been reported by several studies that dysregulation of FGF15/FGF19 signaling contributes to the pathogenesis of several diseases, especially BAs associated metabolic diseases.^[^ ^40^, ^42^ ^]^ However, our results indicate that excessive hepatic BAs accumulation in response to INH and RIF is independent of intestinal FGF15 level, and conversely, co‐treatment of INH and RIF significantly reduces hepatic FGF1 expression (Figure 1; Figure S1, Supporting Information). More importantly, hepatocyte‐specific *Fgf1* deficiency exacerbated INH and RIF‐induced BAs synthetase transcriptions (Figure 2I). Although the role of hepatic FGF1 in the regulation of BAs is not prominent under steady‐state conditions, it plays a more important role than intestinal FGF15 in maintaining BAs homeostasis under the challenge of INH and RIF. Hepatic FGF1 levels were not regulated by FXR agonists (GW4064 and cholic acid),^[^ ^43^ ^]^ suggesting FGF1 failed to sense the dynamic levels of BA in the body and participate in the negative feedback regulation of BA under physiological conditions. In addition, there may be compensatory effects that prevent *Fgf1* deficiency from affecting BA homeostasis in steady‐state conditions, which may be significantly impaired under INH/RIF stress. All of the above may be reasons why FGF1 plays a crucial role in BA homeostasis under INH/RIF stress, rather than in steady‐state conditions. Besides, hepatic expression profiles of FGFs showed that *Fgf1* was the most predominant expressed *Fgf*, accounting for ≈74.5% of the total FGFs in the liver (Figure S1A, Supporting Information). Therefore, it is reasonable to speculate that despite the presence of other FGFs, the specific reduction in *Fgf1* is sufficient to exacerbate isoniazid and rifampicin‐induced liver injury.

Liver‐enriched HNF4*α* plays a central role in BA homeostasis by regulating of genes involved in BA biosynthesis.^[^ ^32^, ^44^ ^]^ HNF4*α* functions as a crucial transcriptional activator and binds to the promoters of key BAs synthases to initiate their basal transcription levels. Here, we found that INH/RIF treatment led to an obvious increase in HNF4*α* in *Fgf1* ^fl/fl^ mice, which may be related to the decreased hepatic FGF1 or directly triggered by INH/RIF. More importantly, under INH/RIF challenge, hepatocyte‐specific *Fgf1* deficiency further elevated hepatic HNF4*α* expression (Figure 4C–E), while FGF1^ΔHBS^ treatment markedly suppressed HNF4*α* levels in vitro and in vivo (Figure 4F,G; Figure S6, Supporting Information). Liver‐specific knockdown of HNF4*α* significantly abolished the BA regulatory activity and hepatoprotective effect of FGF1^ΔHBS^ in INH and RIF‐induced liver injury mouse model (Figure 4K–S). In this study, we found that the inhibition of HNF4*α* was dependent on FGF1‐induced FGFR4‐ERK1/2 activation (Figures 5 and 6; Figure S7, Supporting Information), which is consistent with previous reports that ERK1/2 signaling activation mediates the inhibition of HNF4*α* transcription.^[^ ^45^, ^46^ ^]^ However, to date, the direct molecular link between ERK1/2 activation and the transcriptional regulation of HNF4*α* is still unclear. It is possible that ERK1/2 phosphorylates specific transcription factors known to regulate gene expression of HNF4*α*. Taken together, all these data indicate that FGFR4‐ERK1/2‐HNF4*α* signaling plays a critical role in FGF1 mediated BAs homeostasis.

Our findings in the mouse model suggest that FGF1‐based therapeutic strategies have the potential to be applied in patients with INH/RIF‐induced liver injury. However, several issues remain to be addressed. First, more clinically relevant experiments, such as primary hepatocytes derived from patients with anti‐TB drug‐induced liver injury, and advanced animal models (e.g., primates) are needed to validate our findings. Second, the safety of FGF1‐based therapies should be carefully assessed, mainly due to the widespread distribution of FGFRs and their co‐receptor heparan sulfate (HS) glycosaminoglycans in the body, which likely results in the administration of mitogenic paracrine FGF1 activating multi‐organ signaling and proliferation and thus inducing potential toxic side effects. Although the previously engineered FGF1^ΔHBS^ used in this study retained its therapeutic effect against anti‐TB drug‐induced liver injury without inducing hepatic proliferation, the safety evaluation of FGF1^ΔHBS^ in vivo should be systematically investigated in the future, since the heparin site mutation did not affect its FGFR binding selectivity. In addition, it may be possible to design FGF1 analogs that preferentially activate FGFR4 within a certain threshold range, or to apply liver‐targeted delivery strategies to minimize off‐target effects and adverse outcomes.

In summary, this study demonstrated that the significant reduction of hepatic FGF1 in response to INH and RIF leads to aberrant accumulation of hepatic BAs, ultimately causing drug‐induced liver injury, by inducing FGFR4‐ERK1/2 mediated HNF4*α* expression, which triggers the transcription of key enzymes for BAs biosynthesis (Figure 6O). Our study provided novel mechanistic insights into the pathogenesis of ATB‐DILI and potential therapeutic strategies for the intervention and treatment of this disease.

## 4 Experimental Section

### Animals

All animal experiments and methods performed in this study followed ethical guidelines for animal studies and were approved by the Institutional Animal Care and Use Committee of Wenzhou Medical University, China. Adult (8‐week‐old) male C57BL/6J mice were obtained from Beijing Vital River Laboratory Animal Technology Company. Whole‐body knockout mice of *Fgf1* (*Fgf1* ^KO^), *Fgf1* ^fl/fl^ and *Fgfr4* ^fl/fl^) mice on C57BL/6J background were structured by Cyagen Biosciences. Hepatocyte‐specific *Fgf1* or *Fgfr4* knockout mice (*Fgf1*‐LKO or *Fgfr4*‐LKO) or were generated by crossing albumin‐cre (Alb‐Cre) recombinase transgenic mice with *Fgf1* ^fl/fl^ or *Fgfr4* ^fl/fl^. The mice were housed in a controllable and standard laboratory environment (temperature 20 ± 2 °C, relative humidity 55 ± 10%, and a 12 h light/12 h dark cycle) with water and a chow diet available ad libitum. Genotypes of the transgenic mice were detected by polymerase chain reaction (PCR) analysis using the DNA from the mouse tails, the primers are listed in Table S1 (Supporting Information).

### Human Liver Samples

Human liver pathology sections were collected from The First Affiliated Hospital of Wenzhou Medical University (Zhejiang, China). All research was approved by the ethics committee of The First Affiliated Hospital of Wenzhou Medical Univeristy (the approval number is KY2024‐R147) and conducted in accordance with both the Declarations of Helsinki and Istanbul. The details of human patient information is listed in Table S2 (Supporting Information).

### Adeno‐Associated Viruses (AAVs)

In vivo *Hnf4α* knockdown was achieved via AAV8 vectors. Recombinant adeno‐associated virus serotype 8 vectors carrying *Hnf4α* with a TBG promoter (AAV8‐TBG‐shRNA *Hnf4α*, sh*Hnf4α*) and the adenoviral vector without carrying *Hnf4α* (AAV8‐TBG‐shRNA NC, shNC) were purchased from GeneChem Company. These adenoviral vectors were injected into mice (2 × 10^11^ vector genomes/mouse) to knockdown *Hnf4α* via tail vein injection for 3 weeks. The expression of HNF4*α* was analyzed by western blotting analysis and qRT‐PCR.

### Induction of Anti‐Tuberculosis Drug‐Induced Liver Injury (ATB‐DILI) Mouse Model and Treatment

Eight‐week‐old male mice was intragastrically administered INH and RIF (135mg kg^−1^ INH and 270mg kg^−1^ RIF were dissolved with 0.5% CMC‐Na) or 0.5% CMC‐Na as vehicle daily for 3 weeks to induce anti‐tuberculosis drug‐induced liver injury (ATB‐DILI) mouse model. To investigate the therapeutic effect of FGF1^ΔHBS^ on ATB‐DILI, mice challenged with INH and RIF were intraperitoneally (i.p.) injected with 0.1 mg kg^−1^ body weight (BW) daily for three weeks. At the end of these experiments, liver tissues and serum samples were collected for analysis.

### Culture and Treatment of Cells

The human hepatocellular carcinoma (HepG2) cell line was obtained from American Type Culture Collection. HepG2 cells were cultured in MEM*α* medium supplemented with 10% fetal bovine serum (FBS) and 1% Penicillin–Streptomycin, then incubated in a humidified atmosphere of 5% CO_2_ at 37 °C, and passaged every 2 days by trypsinization.

To mimic ATB‐DILI, HepG2 cells were seeded at an appropriate density in six well plates for 24h and then exposed to 100 µg mL^−1^ INH and 200 µg mL^−1^ RIF (dissolved with DMSO) with PBS or 1000 ng mL^−1^ FGF1^ΔHBS^ for 6 h. Pre‐treated HepG2 cells with 10 µM of U0126 (MedChem Express) for 1 h to selectively inhibit the MEK1 and MEK2 signaling pathways.

### ShRNA Transfection

Short hairpin RNA (shRNA) was used to knockdown *Hnf4α* according to the manufacturer's protocol. ShRNA was produced by GeneChem (Shanghai, China) shRNA target sequences were as follows: 5′‐ACATGTACTCCTGCAGATT‐3′. Briefly, HepG2 cells were seeded at a density of 1 × 10^5^ cells/well in 6‐well plates, then *Hnf4α*‐shRNA was transfected with using Lipofectamine 3000 transfection reagent (ThermoFisher) in Opti‐MEM medium (GIBCO) for 6 h. Subsequently, cells were incubated with normal medium for 48 h to determine the knockdown efficiency.

### Mouse Primary Hepatocyte Isolation and Culture

Hepatocytes were isolated from mice by in situ digestion of the liver with perfusion of type IV collagenase. Briefly, liver tissues were perfused with EGTA solution (5 mM HEPES pH = 7.4, 5 mM glucose, 120 mM NaCl, 4.8 mM KCl, 23.8 mM NaHCO_3_, 1.2 mM KH_2_PO_4_, 1.2 mM MgSO_4_ and 1mM EGTA) into the inferior vena cava. After perfusion, the liver tissues were dissociated into hepatocytes using collagenase solution (5 mM HEPES pH = 7.4, 120 mM NaCl, 4.8 mM KCl, 23.8 mM NaHCO_3_, 1.2 mM KH_2_PO_4_, 1.2 mM MgSO_4_ supplemented with 0.0857 U mL^−1^ type IV collagenase (Roche Diagnostics, Indianapolis, IN) and 5 mM CaCl2). Subsequently, the isolated hepatocytes were washed with PBS (Gibco) and suspended in William's E medium supplemented with 10% (w/v) fetal bovine serum (FBS) (TransGen Biotech) and 1% Penicillin‐Streptomycin (Gibco). Cell viability was assessed by the trypan blue exclusion test. Isolated hepatocytes were seeded at a density of 3.5 × 10^5^ cells/dish in 6‐well plates and maintained at 37 °C in 5% CO_2_. After cell adherence, the fresh media (William's E medium supplemented with 1% bovine serum albumin and 1% Penicillin‐Streptomycin) were used to starve hepatocytes for 6 h. To investigate the direct effects of INH and RIF in vitro, primary hepatocytes from C57BL/6J mice were stimulated with DMSO or different concentrations of INH and RIF (low‐INH+RIF: 4 µg mL^−1^ INH and 8 µg mL^−1^ RIF; medium‐INH+RIF: 20 µg mL^−1^ INH and 40 µg mL^−1^ RIF; high‐INH+RIF: 100 µg mL^−1^ INH and 200 µg mL^−1^ RIF) for 6 h. Pre‐treated Primary hepatocyte with 10 µM U0126 for 1 h selectively inhibits the MEK1 and MEK2 signaling pathways, followed by treated with PBS or 1000 ng mL^−1^ FGF1^ΔHBS^ for 6 h.

### Determination of Biochemical Indexes

Serum alanine aminotransferase (ALT) and aspartate aminotransferase (AST) were analyzed using biochemical analyzer (Beckman). Hepatic and serum total bile acid (TBA) and serum total bilirubin (TBIL) were analyzed using commercial kits according to the manufacturer's protocols (Nanjing Jiancheng Bioengineer Institute).

### Immunofluorescence Staining

The mouse liver tissue was fixed with 4% paraformaldehyde for 24h, then liver sections from paraffin‐embedded tissues were prepared at 5‐µm thickness. The sections were rehydrated and treated for antigen unmasking. After washing, the samples were blocked with 5% BSA in PBS for 30 min and incubated with primary antibodies at 4 °C overnight. In our studies, the following primary antibodies were used: FGF1 (Proteintech, 17400), FGF15 (Santa Cruz Biotechnology, 514647), Albumin (Proteintech, 66051‐1‐Ig), F4/80 (Proteintech, 28463‐1‐AP), Cleaved Caspase 3 (Cell Signaling Technology, 9664S), Ki67 (Abcam, 16667), PCNA (Abcam, 18197), HNF4*α* (Abcam, 181604). After incubating with appropriate secondary antibodies and washing with PBS, the nuclei was stained with DAPI for 15 min. Images were captured with a confocal laser scanning microscope (Nicon). The positive cells and nuclei in a field were counted, and data were expressed as a percentage of positive cells to the nucleus.

### TUNEL Staining

The liver sections were stained with DeadEnd Fluorometric TUNEL System (Promega, G3250) according to the manufacturer's protocol. DAPI was used for nuclei staining. Images were visualized and captured with a confocal laser scanning microscope (Nikon). The number of nucleus and TUNEL‐positive cells per field was counted, and the percentage of TUNEL‐positive cells was calculated.

### Dihydroethidium Staining

After 24 h of fixation with 4% paraformaldehyde, the mouse liver tissue was first soaked with 15% sucrose solution for 12 h at room temperature, then it soaked with 30% sucrose solution under the same conditions. The frozen sections of 5 µm were made with freezing microtome after freezing and embedding soaked liver tissues with O.C.T compound. The liver sections were incubated with dihydroethidium at 37 °C for 30 min in the dark, and the nuclei were stained with DAPI for 15 min. The staining images were captured with a confocal laser scanning microscope (Nikon). The fluorescence intensity of DHE staining was measured using the Image J software.

### H&E Staining

The harvested liver samples were fixed with 4% paraformaldehyde for 24 h and embedded in paraffin, then liver sections from paraffin‐embedded tissues were prepared at 5‐µm thickness. The sections were deparaffinized and rehydrated by immersion in xylene and ethanol at decreasing concentrations. The rehydrated sections were stained with hematoxylin (ZSGB‐Bio, ZLI‐9610) for 2 min and then immersed in PBS solution for 10 min. Then the sections were stained with eosin (ZSGB‐Bio, ZLI‐9613) for 10 s. The sections were then washed, dehydrated, and sealed with neutral resin. Images were captured using a microscope.

### Western Blotting Analysis

Total protein from liver homogenates or cells (HepG2 cell or primary hepatocyte) was extracted with protein extraction reagent (Boster Bioengineering), and nuclear proteins were extracted with Nuclear and Cytoplasmic Protein Extraction Kit (KeyGen BioTech). After the protein concentration in the supernatants was measured using Coomassie brilliant blue method, the protein samples were mixed with protein loading buffer (Fudebio‐tech), then denatured at 100 °C for 10 min. Equal amounts of total protein were separated via 8–15% SDS‐PAGE and transferred to PVDF Membrane (Millipore). Following incubation with TBST‐buffered saline containing 5% bovine serum albumin (BSA) for 1 h at room temperature, membranes incubated overnight at 4 °C with primary antibodies, then washed with TBST and incubated with appropriate secondary antibodies for 1 h at room temperature. Protein bands were identified with an Easysee Western Blot Kit (TransGen Biotech) and visualized using a chemiDoc XRS+ (BioRAD). Densitometer analysis was performed using the ImageLab analysis software. The following antibodies were used in western blot: FGF1 (Proteintech, 17400), GAPDH (Cell Signaling Technology, 2118S), Cyp27a1 (Abcam, 126785), Cyp7a1 (Abcam, 65596), Cyp8b1 (Abcam, 191910), pFGFR4 (Abcam, 192589), FGFR4 (Proteintech, 11098‐1‐AP), HNF4*α* (Abcam, 181604), p‐ERK1/2 (Cell Signaling Technology, 4370S), ERK1/2 (Cell Signaling Technology, 9102S), p‐PI3K (Cell Signaling Technology, 4228S), p‐AKT (Proteintech, 66444), AKT (Cell Signaling Technology, 4691S), p‐JNK (Cell Signaling Technology, 4668S), JNK (Cell Signaling Technology, 9252S).

### RNA Isolation and Quantitative Real‐Time‐PCR (qRT‐PCR)

Total RNA was extracted from mouse liver tissue and other tissues using Trizol reagent (TransGen Biotech), as described by the manufacturer's instructions. The RNA samples were reversely transcribed to cDNA by the TransScript Reverse Transcriptase kit (TransGen Biotech). Analysis of qRT‐PCR was performed on a Roche LightCycler 480 II (Roche) RealTime PCR Detection System using Taq Pro Universal SYBR qPCR Master Mix (Vazyme) with specific primers. The relative expression levels of target gene were quantitated using the 2−ΔΔCT method and normalized to the amount of *β*‐Actin. The sequences of specific primers used for qRT‐PCR in this study are listed in Table S3 (Supporting Information).

### ELISA Assays

ELISA assays were performed to evaluate the serum FGF1 (Wuhan USCN Business, SEA032Hu), FGF15 (Quanzhou Konodi Biotechnology, SU‐B21152) and FGF19 (Wuhan USCN Business, SEC917Hu) levels in mouse and human according to the manufacturer's instructions. The supernatant of primary hepatocytes was collected, then centrifuged at 100 xg for 20 min, and FGF1 concentrations were determined using an FGF1 ELISA kit (Wuhan USCN Business, SEA032Hu).

### Statistical Analysis

All experimental results were expressed as the mean ± SEM. Statistical analyses were done using GraphPad Prism 8 (GraphPad Software). The statistical comparisons between two experimental groups were conducted using a two‐tailed students’ ^t^‐test. The differences between multiple groups were analyzed using ordinary one‐way analysis of variance (ANOVA) or two‐way AVOVA. P < 0.05 was considered statistically significant.

## Conflict of Interest

The authors declare no conflict of interest.

## Author Contributions

Q.L. and J.Z. contributed equally to this work. J.W. and J.N. conceived the project and designed experiments. Q.L., J.Z., J.Q., J.T., S.C., S.Z., X.L., H.L., J.L., R.L., J.X., Y.W., L.Y., J.W., and J.N. performed experiments, analyzed data and participated in discussion of the results. Y.J. provided human pathological tissue slices. Q.L., L.Y., J.W., and J.N. wrote this manuscript.

## Supporting information



Supporting Information

## Data Availability

The data that support the findings of this study are available from the corresponding author upon reasonable request.

## References

[1] B. E. Senousy , S. I. Belal , P. V. Draganov , Nat. Rev. Gastroenterol. Hepatol. 2010, 7, 543. 20808293 10.1038/nrgastro.2010.134

[2] W. B. Park , W. Kim , K. L. Lee , J.‐J. Yim , M. Kim , Y. J. Jung , N. J. Kim , D. H. Kim , Y. J. Kim , J.‐H. Yoon , M.‐D. Oh , H. S. Lee , J. Infect. 2010, 61, 323. 20670648 10.1016/j.jinf.2010.07.009

[3] W. S. Lim , A. Avery , O. M. Kon , M. Dedicoat , BMJ 2023, 383, e074866. 37890885 10.1136/bmj-2023-074866

[4] G. P. Aithal , P. B. Watkins , R. J. Andrade , D. Larrey , M. Molokhia , H. Takikawa , C. M. Hunt , R. A. Wilke , M. Avigan , N. Kaplowitz , E. Bjornsson , A. K. Daly , Clin. Pharmacol. Ther. 2011, 89, 806. 21544079 10.1038/clpt.2011.58

[5] P. Ichai , F. Saliba , F. Antoun , D. Azoulay , M. Sebagh , T. M. Antonini , L. Escaut , D. Valérie , D. Castaing , D. Samuel , Liver Transpl. 2010, 16, 1136. 20879012 10.1002/lt.22125

[6] R. Kumar , B. V. Shalimar , S. Khanal , V. Sreenivas , S. D. Gupta , S. K. Panda , S. K. Acharya , Hepatology 2010, 51, 1665. 20196116 10.1002/hep.23534

[7] X. Zhuang , L. Li , T. Liu , R. Zhang , P. Yang , X. Wang , L. Dai , Front. Pharmacol. 2022, 13, 1037814. 36299895 10.3389/fphar.2022.1037814 PMC9589499

[8] I. Metushi , J. Uetrecht , E. Phillips , Br. J. Clin. Pharmacol. 2016, 81, 1030. 26773235 10.1111/bcp.12885 PMC4876174

[9] S. A. Tasduq , P. Kaiser , S. C. Sharma , R. K. Johri , Hepatol. Res. 2007, 37, 845. 17573957 10.1111/j.1872-034X.2007.00129.x

[10] L. Liu , X. Li , C. Huang , Y. Bian , X. Liu , J. Cao , W. Qu , L. Miao , Expert Opin. Drug Metab. Toxicol. 2020, 16, 527. 32436768 10.1080/17425255.2020.1758060

[11] F. Li , J. Lu , J. Cheng , L. Wang , T. Matsubara , I. L. Csanaky , C. D. Klaassen , F. J. Gonzalez , X. Ma , Nat. Med. 2013, 19, 418. 23475203 10.1038/nm.3104 PMC3618537

[12] C. Enriquez‐Cortina , M. Almonte‐Becerril , D. Clavijo‐Cornejo , M. Palestino‐Domínguez , O. Bello‐Monroy , N. Nuño , A. López , L. Bucio , V. Souza , R. Hernández‐Pando , L. Muñoz , M. C. Gutiérrez‐Ruiz , L. E. Gómez‐Quiroz , Toxicol. Sci. 2013, 135, 26. 23764483 10.1093/toxsci/kft134

[13] A. Chowdhury , A. Santra , K. Bhattacharjee , S. Ghatak , D. R. Saha , G. K. Dhali , J. Hepatol. 2006, 45, 117. 16545483 10.1016/j.jhep.2006.01.027

[14] Y. Deng , X. Luo , X. Li , Y. Xiao , B. Xu , H. Tong , Front. Pharmacol. 2022, 13, 925509. 35754491 10.3389/fphar.2022.925509 PMC9226894

[15] Y. Wen , G. Zhang , X. Wu , Toxicology 2022, 476, 153256. 35835356 10.1016/j.tox.2022.153256

[16] A. Beenken , M. Mohammadi , Nat. Rev. Drug. Discov. 2009, 8, 235. 19247306 10.1038/nrd2792 PMC3684054

[17] A. Zinkle , M. Mohammadi , F1000Res 2018, 7, 872. 10.12688/f1000research.14143.1 PMC6013765 29983915

[18] R. Goetz , M. Mohammadi , Nat. Rev. Mol. Cell Biol. 2013, 14, 166. 23403721 10.1038/nrm3528 PMC3695728

[19] L. Song , L. Wang , Y. Hou , J. Zhou , C. Chen , X. Ye , W. Dong , H. Gao , Y.i Liu , G. Qiao , T. Pan , Q. Chen , Y.u Cao , F. Hu , Z. Rao , Y. Chen , Y. Han , M. Zheng , Y. Luo , X. Li , Y. Chen , Z. Huang , Hepatology 2022, 76, 1105. 35152446 10.1002/hep.32404

[20] L. Geng , K. S. L. Lam , A. Xu , Nat. Rev. Endocrinol. 2020, 16, 654. 32764725 10.1038/s41574-020-0386-0

[21] C. Degirolamo , C. Sabba , A. Moschetta , Nat. Rev. Drug. Discov. 2016, 15, 51. 26567701 10.1038/nrd.2015.9

[22] L. Jin , R. Yang , L. Geng , A. Xu , Annu. Rev. Pharmacol. Toxicol. 2023, 63, 359. 36100222 10.1146/annurev-pharmtox-032322-093904

[23] R. M. Gadaleta , A. Moschetta , Nat. Metab. 2019, 1, 588. 32694803 10.1038/s42255-019-0074-3

[24] R. Loomba , A. J. Sanyal , K. V. Kowdley , D. L. Bhatt , N. Alkhouri , J. P. Frias , P. Bedossa , S. A. Harrison , D. Lazas , R. Barish , M. D. Gottwald , S. Feng , G. D. Agollah , C. L. Hartsfield , H. Mansbach , M. Margalit , M. F. Abdelmalek , N. Engl. J. Med. 2023, 389, 998. 37356033 10.1056/NEJMoa2304286 PMC10718287

[25] Q. Lin , Z. Huang , G. Cai , X. Fan , X. Yan , Z. Liu , Z. Zhao , J. Li , J. Li , H. Shi , M. Kong , M.‐H. Zheng , D. J. Conklin , P. N. Epstein , K. A. Wintergerst , M. Mohammadi , L. Cai , X. Li , Y. Li , Y. Tan , Hepatology 2021, 73, 2206. 32965675 10.1002/hep.31568 PMC8082952

[26] R. Chen , J. Wang , Y. Zhang , S. Tang , S. Zhan , Arch. Toxicol. 2015, 89, 883. 25693865 10.1007/s00204-015-1473-1

[27] D. Ezhilarasan , Drug Metab. Rev. 2023, 55, 239. 37218081 10.1080/03602532.2023.2215478

[28] Z. Huang , Y. Tan , J. Gu , Y. Liu , L. Song , J. Niu , L. Zhao , L. Srinivasan , Q. Lin , J. Deng , Y. Li , D. J. Conklin , T. A. Neubert , L. Cai , X. Li , M. Mohammadi , Cell Rep. 2017, 20, 1717. 28813681 10.1016/j.celrep.2017.06.063 PMC5821125

[29] S. J. Karpen , J. Hepatol. 2002, 36, 832. 12044537 10.1016/s0168-8278(02)00129-0

[30] D. W. Russell , Annu. Rev. Biochem. 2003, 72, 137. 12543708 10.1146/annurev.biochem.72.121801.161712

[31] G. Zollner , H. U. Marschall , M. Wagner , M. Trauner , Mol. Pharm. 2006, 3, 231. 16749856 10.1021/mp060010s

[32] Y. Song , C. Xu , S. Shao , J. Liu , W. Xing , J. Xu , C. Qin , C. Li , B. Hu , S. Yi , X. Xia , H. Zhang , X. Zhang , T. Wang , W. Pan , C. Yu , Q. Wang , X. Lin , L. Wang , L. Gao , J. Zhao , J. Hepatol. 2015, 62, 1171. 25533663 10.1016/j.jhep.2014.12.006

[33] S. Kir , Y. Zhang , R. D. Gerard , S. A. Kliewer , D. J. Mangelsdorf , J. Biol. Chem. 2012, 287, 41334. 23038264 10.1074/jbc.M112.421834 PMC3510831

[34] Y. Xie , N. Su , J. Yang , Q. Tan , S. Huang , M. Jin , Z. Ni , B. Zhang , D. Zhang , F. Luo , H. Chen , X. Sun , J. Q. Feng , H. Qi , L. Chen , Signal Transduct Target Ther. 2020, 5, 181. 32879300 10.1038/s41392-020-00222-7 PMC7468161

[35] H. Kurosu , M. Choi , Y. Ogawa , A. S. Dickson , R. Goetz , A. V. Eliseenkova , M. Mohammadi , K. P. Rosenblatt , S. A. Kliewer , M. Kuro‐o , J. Biol. Chem. 2007, 282, 26687. 17623664 10.1074/jbc.M704165200 PMC2496965

[36] C. D. Fuchs , M. Trauner , Nat. Rev. Gastroenterol. Hepatol. 2022, 19, 432. 35165436 10.1038/s41575-021-00566-7

[37] Y. Pan , H. Zhang , M. Li , T. He , S. Guo , L. Zhu , J. Tan , B. Wang , Gut Microbes 2024, 16, 2356284. 38769683 10.1080/19490976.2024.2356284 PMC11110704

[38] M. Trauner , C. D. Fuchs , E. Halilbasic , G. Paumgartner , Hepatology 2017, 65, 1393. 27997980 10.1002/hep.28991

[39] J. P. Arab , S. J. Karpen , P. A. Dawson , M. Arrese , M. Trauner , Hepatology 2017, 65, 350. 27358174 10.1002/hep.28709 PMC5191969

[40] T. Inagaki , M. Choi , A. Moschetta , L. Peng , C. L. Cummins , J. G. McDonald , G. Luo , S. A. Jones , B. Goodwin , J. A. Richardson , R. D. Gerard , J. J. Repa , D. J. Mangelsdorf , S. A. Kliewer , Cell Metab. 2005, 2, 217. 16213224 10.1016/j.cmet.2005.09.001

[41] K. H. Song , T. Li , E. Owsley , S. Strom , J. Y. Chiang , Hepatology 2009, 49, 297. 19085950 10.1002/hep.22627 PMC2614454

[42] X. Li , W. Lu , A. Kharitonenkov , Y. Luo , J. Intern. Med. 2024, 295, 292. 38212977 10.1111/joim.13767 PMC13574229

[43] L. Song , Y. Hou , D. Xu , X. Dai , J. Luo , Y. Liu , Z. Huang , M. Yang , J. Chen , Y. Hu , C. Chen , Y. Tang , Z. Rao , J. Ma , M. Zheng , K. Shi , C. Cai , M. Lu , R. Tang , X. Ma , C. Xie , Y. Luo , X. Li , Z. Huang , Cell Metab. 2024.

[44] Y. Xu , Y. Zhu , S. Hu , Y. Xu , D. Stroup , X. Pan , F. C. Bawa , S. Chen , R. Gopoju , L. Yin , Y. Zhang , Hepatology 2021, 73, 2251. 33098092 10.1002/hep.31604 PMC8062586

[45] Z. Xu , O. L. Tavares‐Sanchez , Q. Li , J. Fernando , C. M. Rodriguez , E. J. Studer , W. M. Pandak , P. B. Hylemon , G. Gil , J. Biol. Chem. 2007, 282, 24607. 17603092 10.1074/jbc.M611481200 PMC3291957

[46] M. Dezhbord , S. H. Kim , S. Park , D. R. Lee , N. Kim , J. Won , A. R. Lee , D.‐S. Kim , K.‐H. Kim , Clin. Mol. Hepatol. 2024, 30, 1060. 39044466 10.3350/cmh.2024.0060e PMC11540370

