# Supplementary material for: Hepatocyte‐Derived FGF1 Alleviates Isoniazid and Rifampicin‐Induced Liver Injury by Regulating HNF4α‐Mediated Bile Acids Synthesis
Source: Adv Sci (Weinh). 2024 Dec 27;12(7):2408688. doi: 10.1002/advs.202408688 (PMC11831436; doi:10.1002/advs.202408688)
Supplement: Supplementary file 1 — Supporting Information [file ADVS-12-2408688-s001.docx]

**Supporting Information**

**Hepatocyte-derived FGF1 Alleviates Isoniazid and Rifampicin-induced Liver Injury by Regulating HNF4α-mediated Bile Acids Synthesis**

Qian Lin ^1, #^, Jiaren Zhang ^1, #^, Jie Qi ^1^, Jialing Tong ^1^, Shenghuan Chen ^1^, Sudan Zhang ^1^, Xingru Liu ^1^, Huatong Lou ^1^, Jiaxuan Lv ^1^, Ruoyu Lin ^1^, Junjun Xie ^2^, Yi Jin ^3^, Yang Wang ^4^, Lei Ying ^4,^ *, Jiamin Wu ^1,^ *, Jianlou Niu ^1,^ *

^1^ School of Pharmaceutical Sciences, Wenzhou Medical University, Wenzhou 325035, Zhejiang, China.

^2^ Sir Run Run Shaw Hospital, School of Medicine, Zhejiang University, Hangzhou, 310016, Zhejiang, China.

^3^ Department of Pathology, The First Affiliated Hospital of Wenzhou Medical University, Wenzhou 325035, Zhejiang, China.

^4^ School of Basic Medical Sciences, Wenzhou Medical University, Wenzhou, Zhejiang 325035, China.

^#^ These authors contributed equally to this work.

* To whom correspondence should be addressed:

Jianlou Niu, School of Pharmaceutical Sciences, Wenzhou Medical University, Chashan Town, Wenzhou 325035, China. Tel: +86 13676451764; Email: [niujianlou@wmu.edu.cn](mailto:niujianlou@126.com).

Jiamin Wu, School of Pharmaceutical Sciences, Wenzhou Medical University, Chashan Town, Wenzhou 325035, China. Tel: +86 15057728975; Email: wujiamin@yeah.net.

Lei Ying, School of Basic Medical Sciences, Wenzhou Medical University, Wenzhou, Chashan Town, Zhejiang 325035, China. Tel: +86 15058717268; Email: leiying_wmu@wmu.edu.cn.


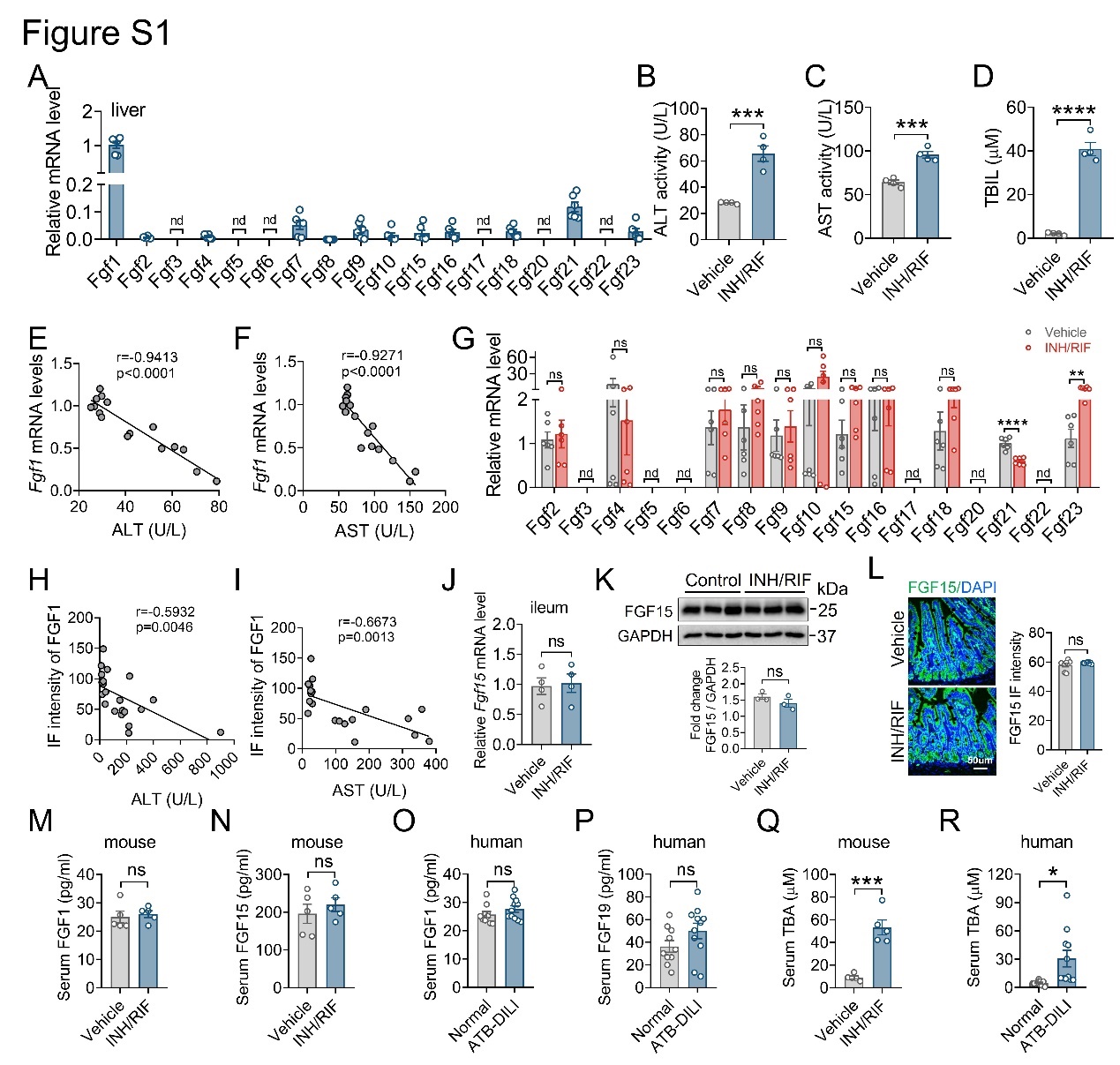


**Figure S1 Construction and analysis of INH and RIF-induced liver injury**

(A) The expression profile of all hepatic *Fgfs* in normal C57BL/6J mice was detected by qRT-PCR and normalized to *β-Actin* (n=6).

(B-D) Eight-week-old male C57BL/6J mice were challenged with 135 mg/kg isoniazid and 270 mg/kg rifampicin or vehicle by gavage for three weeks. At the end of this experiment, serum samples were collected for analysis (n=4). Serum ALT (B), AST (C) and TBIL (D) levels were analyzed in vehicle and INH/RIF-treated mice.

(E-F) The correlation between hepatic *Fgf1* mRNA level and serum ALT (E) or AST (F) level in vehicle and INH/RIF-treated mice (n=16).

(G) The mRNA levels of all *Fgfs,* expect *Fgf1*, in primary hepatocytes from vehicle and INH/RIF-treated mice were analyzed by qRT-PCR and normalized to *β-Actin* (n=6).

(H-I) Correlation analyses of FGF1 levels against serum activities of ALT (H) and AST (I) in human patients challenged with INH and RIF (n=21).

(J-L) The expression level of intestinal *Fgf15* in vehicle and INH/RIF-treated mice was analyzed by qRT-PCR (J), western blotting analysis (K) and immunofluorescence staining (L). The relative *Fgf15* mRNA level was normalized to that of *β-Actin* (n=4). The relative FGF15 protein expression level was determined after normalization with GAPDH (n=3). The expression of FGF15 (green) was quantified using Image J software (n=8, two fields per mice). Scale bar = 50 μm.

(M-P) The serum FGF1 and FGF15/19 concentrations were detected by ELISA Kit in both mouse model (M-N) and human patients (O-P) with INH and RIF-induced liver injury.

(Q-R) The serum TBA levels were detected in both mouse model (n=5) (Q) and human patients (n=10-11) (R) with INH and RIF-induced liver injury.

Data are presented as mean ± SEM; (B-D, G, J, lower panel of K and L, M-R) two-tailed unpaired t-test; (E-F, H-I) Spearman’s correlation. ***P < 0.001; **** p < 0.0001; ns, not significant; nd, not detectable.


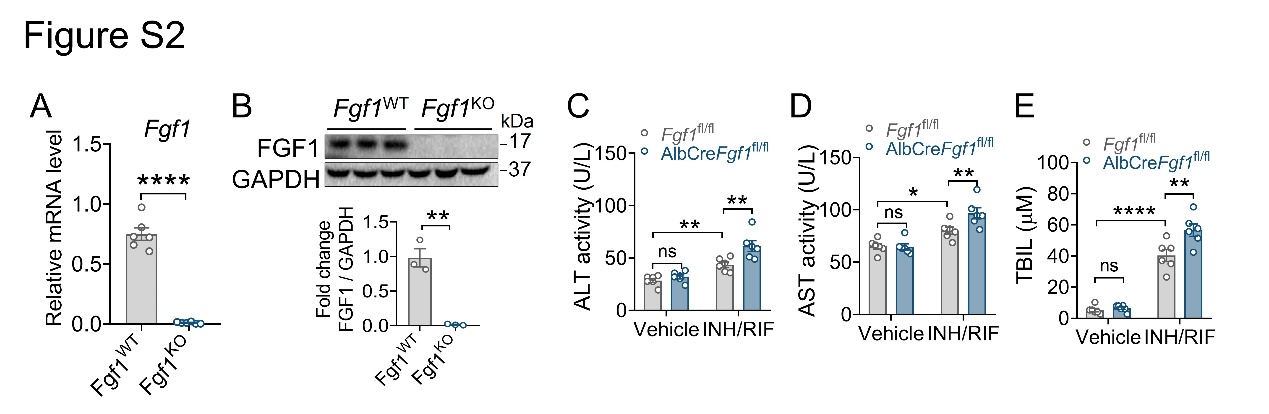


**Figure S2 Knockout of *Fgf1* exacerbated INH and RIF-induced liver injury**

(A-B) Eight-week-old male *Fgf1*^WT^ and *Fgf1*^KO^ mice were challenged with vehicle or 135 mg/kg isoniazid and 270 mg/kg rifampicin by gavage for three weeks. Liver tissues and serum samples were collected at the end of this experiment. Verification of *Fgf1* knockout was detected by qRT-PCR (A) and western blotting analysis (B). The relative *Fgf1* mRNA level was normalized to that of *β-Actin* (n=6). The relative FGF1 protein expression level was determined after normalization with GAPDH (n=3).

(C-E) Serum ALT (C), AST (D), serum TBIL contents (E) (n=6).

Data are presented as mean ± SEM; (A-B) two-tailed unpaired t-test; (C-E) ordinary two-way ANOVA, followed by Sidak. *P < 0.05; **P < 0.01; **** p < 0.0001.


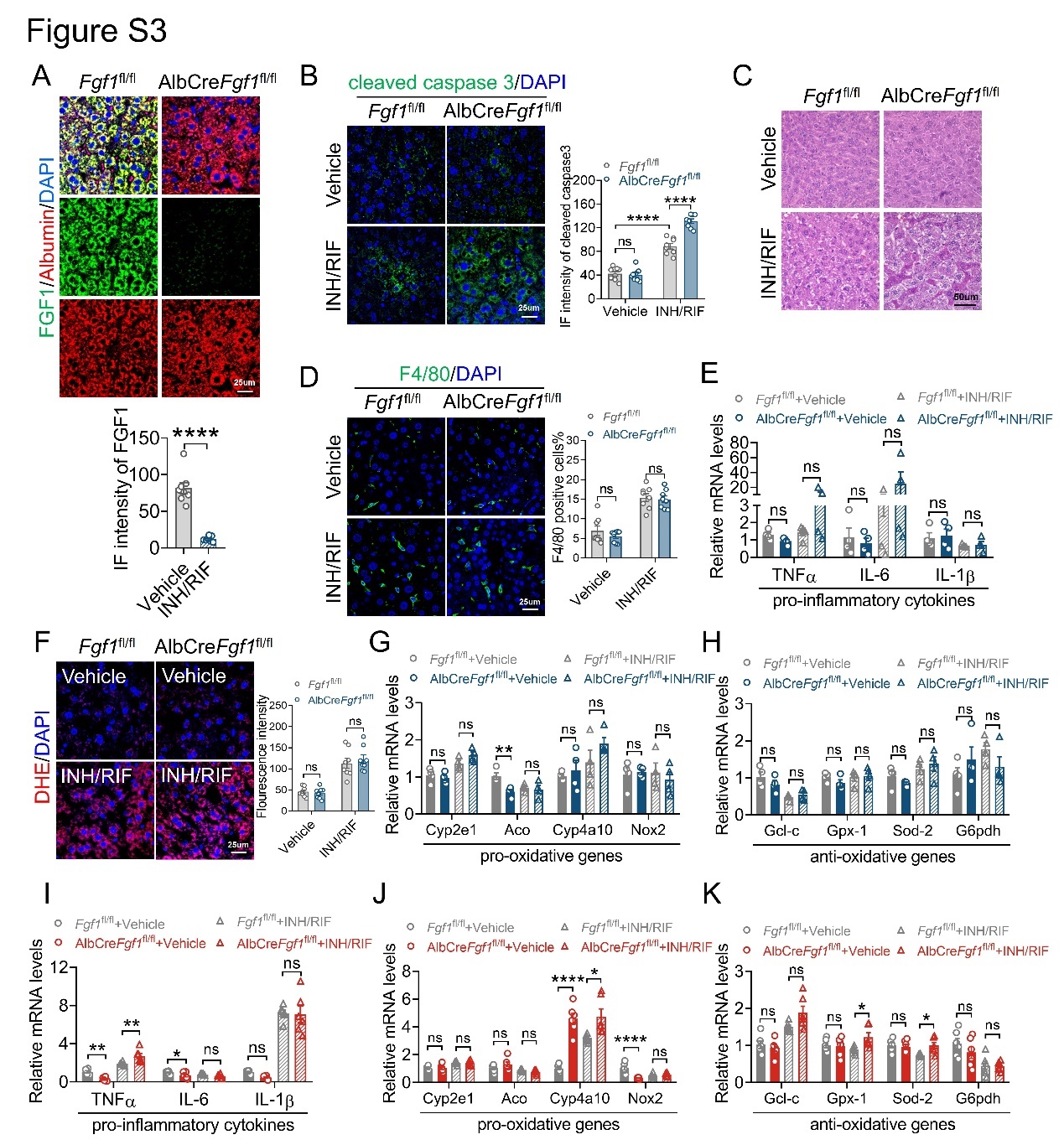


**Figure S3 Hepatocyte-specific *Fgf1* deficiency did not change the levels of oxidative stress or inflammation *in vivo* and *in vitro***

(A) The expression of FGF1 (green) and hepatocyte biomarker albumin (red) was detected by immunofluorescence staining and quantified using Image J software (n=8, two fields per mice). Scale bar = 25 μm.

(B-H) Eight-week-old male *Fgf1*^fl/fl^ and *Fgf1*-LKO mice were challenged with 135 mg/kg isoniazid and 270 mg/kg rifampicin or vehicle by gavage for three weeks. Liver tissues were collected at the end of this experiment.

(B) Representative image and quantification of liver sections stained with cleaved caspase 3 (green), DAPI (blue) from the vehicle and INH and RIF-treated mice (n=8, two fields per mouse). The cleaved caspase 3 stains were quantified using Image J software. Scale bar = 25 μm.

(C) Representative images of liver sections from the vehicle or INH/RIF-treated *Fgf1*^fl/fl^ and *Fgf1*-LKO mice stained with H&E staining. Scale bar = 50 μm.

(D) Representative image and quantification of liver sections stained with TUNEL (green) and DAPI (blue) from the *Fgf1*^fl/fl^ and *Fgf1*-LKO mice challenged with vehicle or INH/RIF (n=8, fields per mice). Scale bar = 25 μm.

(E) The mRNA levels of pro-inflammatory genes in the livers of the *Fgf1*^fl/fl^ and *Fgf1*-LKO mice challenged with vehicle or INH/RIF were analyzed by qRT-PCR and normalized to that of *β-Actin* (n=4).

(F) Hepatic level of ROS was determined by dihydroethidium (DHE) staining in liver tissues (n=8, two fields per mice). Scale bar=25 μm.

(G-H) The mRNA levels of the pro-oxidant cytokines (G) and anti-oxidative genes (H) in liver tissues were determined by qRT-PCR and normalized to that of *β-Actin* (n=4).

(I-K) The mRNA levels of pro-inflammatory genes (I), pro-oxidant genes (J) and anti-oxidative genes (K) in primary hepatocytes extracted from *Fgf1*^fl/fl^ and *Fgf1*-LKO mice treated with vehicle or INH/RIF were analyzed by qRT-PCR and normalized to that of *β-Actin* (n=6).

Data are presented as mean ± SEM; (lower panel of A) two-tailed unpaired t-test; (right panel of B and D and F, E, G-K) ordinary two-way ANOVA, followed by Sidak. *P < 0.05; **P < 0.01; **** p < 0.0001, ns, not significant.


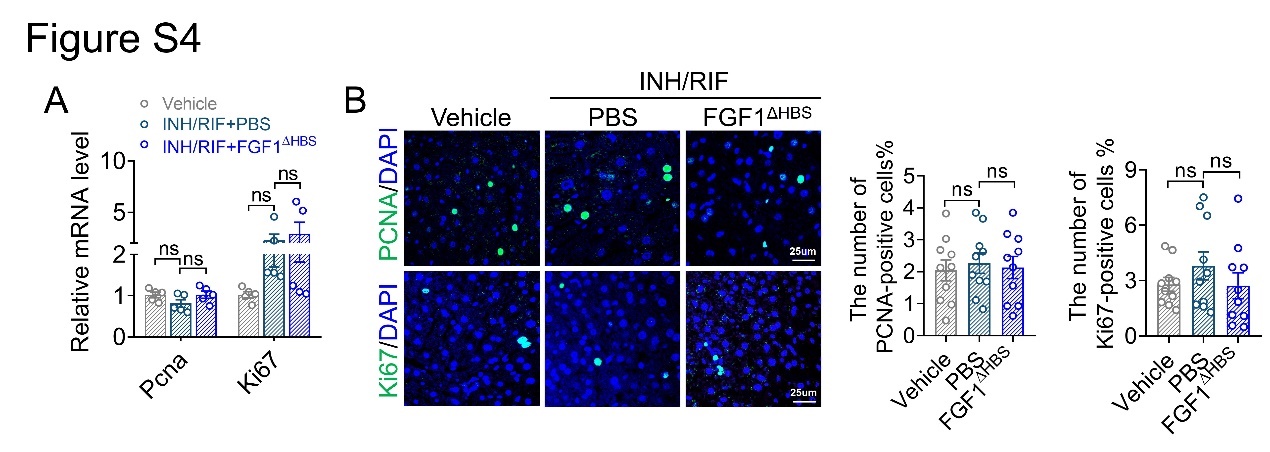


**Figure S4 FGF1^ΔHBS^ did not induce hepatic proliferation in the INH and RIF-induced liver injury mouse model**

(A-B) Eight-week-old male C57BL/6J mice were challenged with 135 mg/kg isoniazid and 270 mg/kg rifampicin or vehicle by gavage, followed by daily i.p. administration of PBS or FGF1^ΔHBS^ (0.1 mg/kg BW/day) for three weeks (n=5).

(A) The hepatic mRNA levels of *Pcna* and *Ki67* were analyzed by qRT-PCR and normalized to *β-Actin*.

(B) Representative image of PCNA (green) and Ki67 (green) of liver sections and quantification by PCNA and Ki67 positive cells (n=10, two fields per mice). Scale bar = 25 μm.

Data are presented as mean ± SEM; (A, right panel of B) ordinary one-way ANOVA, followed by Dunnett. ns, not significant.


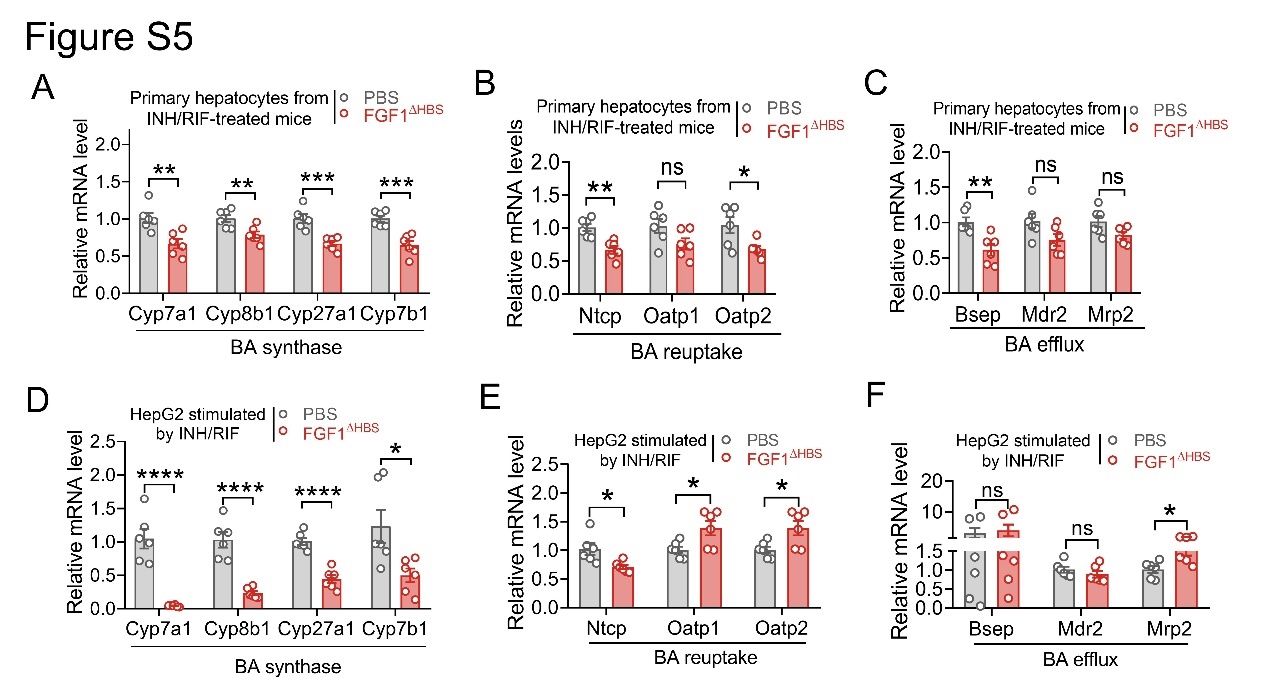


**Figure S5 FGF1^ΔHBS^ treatment did not change the levels of BAs reuptake and efflux transporters**

(A-C) Primary hepatocytes extracted from INH/RIF-treated mice were stimulated with PBS or 1000 ng/ml FGF1^ΔHBS^ for 6 hours. The mRNA levels of BAs synthases (A), BAs transporters of reuptake (B) and efflux (C) were analyzed by qRT-PCR and normalized to *β-Actin* (n=6).

(D-F) HepG2 cells treated with 100 μg/ml INH and 200 μg/ml RIF were stimulated with PBS or 1000 ng/ml FGF1^ΔHBS^ for 6 hours. The mRNA levels of BAs synthases (D), BAs transporters of reuptake (E) and efflux (F) were analyzed by qRT-PCR and normalized to *β-Actin* (n=6).

Data are presented as mean ± SEM; two-tailed unpaired t-test. *P < 0.05; **P < 0.01; ***P < 0.001; ****P < 0.0001; ns, not significant.


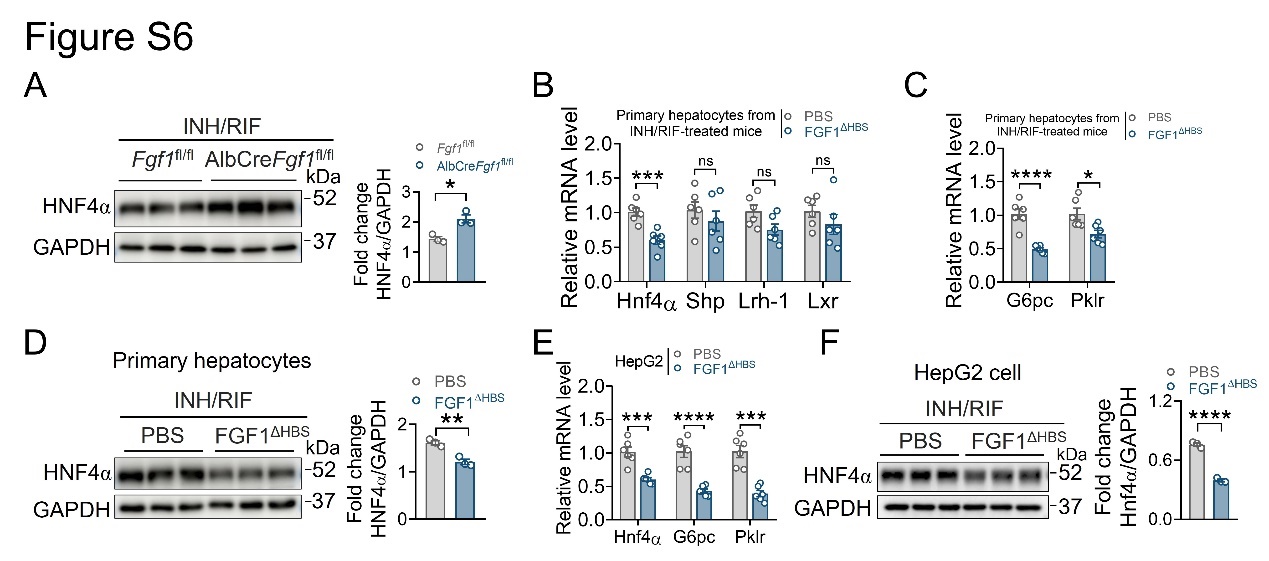


**Figure S6 Effects of FGF1 on the expression levels of HNF4α *in vitro***

(A) The protein level of HNF4α in primary hepatocytes extracted from INH/RIF-treated *Fgf1*^fl/fl^ and AlbCre*Fgf1*^fl/fl^ mice was detected by western blotting analysis and its semi-quantitation using Image J. The relative protein levels were determined after normalization with GAPDH (n=3).

(B-D) Primary hepatocytes extracted from INH/RIF-treated mice were stimulated with PBS or 1000 ng/ml FGF1^ΔHBS^. The mRNA levels of *Hnf4α*, *Shp*, *Lrh-1*, *Lxr* (B) and *Hnf4a* target genes (*G6pc* and *Pklr)* (C) and protein expression of HNF4α (D) were determined by qRT-PCR (normalization with *β-Actin*) (n=6) and western blotting analysis (normalization with GAPDH) (n=3) respectively.

(E-F) HepG2 cells treated with 100 μg/ml INH and 200 μg/ml RIF were stimulated by PBS or 1000 ng/ml FGF1^ΔHBS^. The mRNA levels of *Hnf4α* and its target genes (E) and protein expression of HNF4α were detected by qRT-PCR (normalization with *β-Actin*) (n=6) and western blotting analysis (normalization with GAPDH) (n=3) respectively.

Data are presented as mean values ± SEM; two-tailed unpaired t-test. *P < 0.05; **P < 0.01; ***P < 0.001; **** p < 0.0001, ns, not significant.


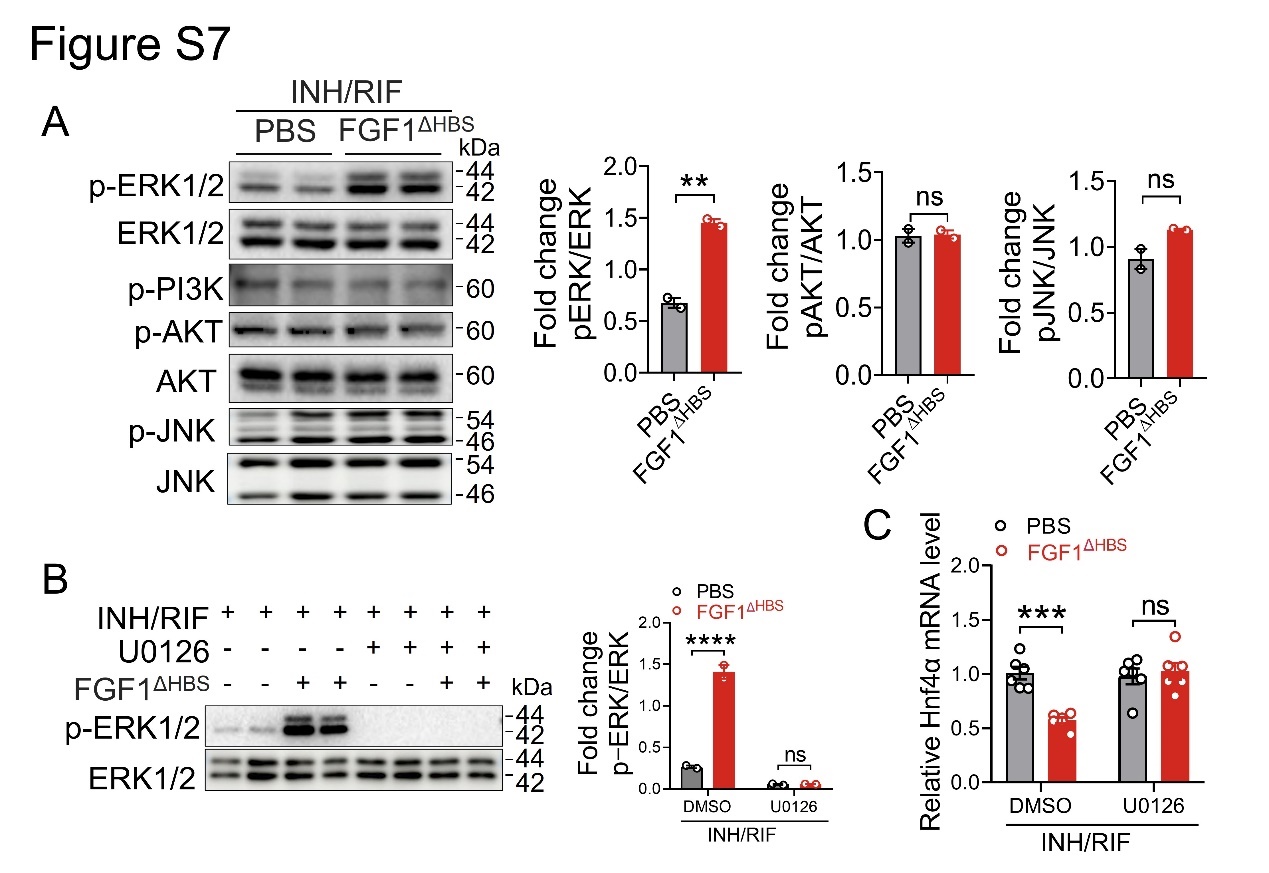


**Figure S7 ERK1/2 partially mediates the inhibitory effect of FGF1^ΔHBS^ on BAs synthase in HepG2 cell**

(A) HepG2 cells treated with 100 μg/ml INH and 200 μg/ml RIF were stimulated by PBS or 1000 ng/ml FGF1^ΔHBS^ for 5 min. Protein levels were determined by western blotting analysis and its semi-quantitation using Image J. The relative protein expression level was determined after normalization with corresponding total protein (n=2).

(B-C) HepG2 cells pretreated with U0126 (10 μM) for 2 hours were challenged with 100 μg/ml INH and 200 μg/ml RIF, followed by stimulation of PBS or 1000 ng/ml FGF1^ΔHBS^ for 6 hours. The protein levels of p-ERK1/2, ERK1/2 were determined by western blotting analysis and its semi-quantitation using Image J (B) (n=2). The mRNA level of *Hnf4α* was determined by qRT-PCR and normalized to *β-Actin* (C) (n=6).

Data are presented as mean values ± SEM; (right panel of A) two-tailed unpaired t-test; (right panel of B, C) ordinary two-way ANOVA, followed by Sidak. **P < 0.01; ***P < 0.001; **** p < 0.0001, ns, not significant.


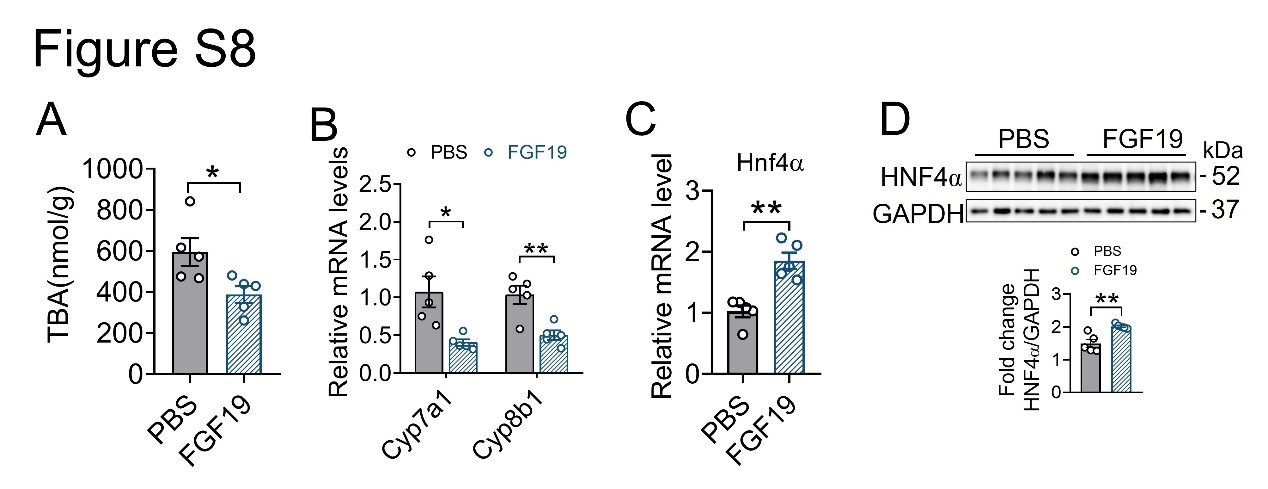


**Figure S8 Recombinant FGF19 regulates bile acid synthase independent on HNF4α under the challenge of INH and RIF**

(A-D) Eight-week-old male C57BL/6J mice challenged with 135 mg/kg isoniazid and 270 mg/kg rifampicin by gavage were daily intraperitoneally (i.p.) injected with recombinant FGF19 (0.1 mg/kg) or PBS for three weeks (n=5).

(A) Hepatic TBA contents in INH/RIF-treated mice injected with recombinant FGF19 or PBS.

(B-C) The mRNA levels of *Cyp7a1*, *Cyp8b1* and *Hnf4α* in liver tissues from INH and RIF-treated mice treated with PBS or FGF19 were analyzed by qRT-PCR and normalized to *β-Actin*.

(D) Hepatic protein expression of HNF4α as determined by western blotting analysis and its semi-quantitation using Image J. The relative protein levels were determined after normalization with GAPDH.

Data are presented as mean values ± SEM; (A-D) two-tailed unpaired t-tes. *P < 0.05; **P < 0.01.

**Supplemental Table S1. Primers of genes used for PCR**

| **Mice** | **Name** | **Forward (5’-3’)** | **Reverse (5’-3’)** |
| --- | --- | --- | --- |
| Fgf1-  KO | Fgf1 | GCTACGGAATAGAGAATGAGCGA | TCTATGAGAGTGAGGTTGGTACTG |
|  | Fgf1  WT | CGAGCACTTATAAGAATCTTGCCT | TCTATGAGAGTGAGGTTGGTACTG |
| Fgf1-  LKO | Fgf1  flox | CGAGAACTCTCTCCTCTCTAGGAA | GAGTACAGCAGTCCTTGTAGAACA |
|  | AlbCre | GAAGCAGAAGCTTAGGAAGATGG | TTGGCCCCTTACCATAACTG |
|  | AlbCre  WT | TGCAAACATCACATGCACAC | TTGGCCCCTTACCATAACTG |
| Fgfr4-LKO | Fgfr4  flox1 | CCCTTACTTCTTAGGGTCGAAGT | TCTAGGTCAGCCTCAAAACAGAAC |
|  | Fgfr4  flox2 | GGTGAGGAGAAACACTGAGCTAC | TCTAAGCCCTGGATTGAGTCTTTCA |
|  | AlbCre | GAAGCAGAAGCTTAGGAAGATGG | TTGGCCCCTTACCATAACTG |
|  | AlbCre  WT | TGCAAACATCACATGCACAC | TTGGCCCCTTACCATAACTG |

**Supplemental Table S2 Clinical-pathological characteristics of anti-tuberculosis DILI patients used in this study**

| **ID** | **Gender** | **Age**  **(Year)** | **History**  **of TB** | **ALT (U/L)** | **AST (U/L)** | | **ALP**  **(U/L)** | **TbiL**  **(μmol/L)** | **DbiL**  **(μmol/L)** | **Medication**  **history** |
| --- | --- | --- | --- | --- | --- | --- | --- | --- | --- | --- |
| 1 | Male | 75 | No | 12 | 22 | 63 | | 20 | 4 | No |
| 2 | Female | 65 | No | 15 | 18 | 72 | | 19 | 4 | No |
| 3 | Male | 69 | No | 25 | 26 | 56 | | 13 | 3 | No |
| 4 | Female | 62 | No | 17 | 29 | 77 | | 9 | 2 | No |
| 5 | Male | 51 | No | 27 | 25 | 48 | | 14 | 3 | No |
| 6 | Female | 69 | No | 57 | 31 | 92 | | 13 | 3 | No |
| 7 | Female | 56 | No | 19 | 30 | 78 | | 12 | 2 | No |
| 8 | Male | 39 | No | 44 | 22 | 77 | | 18 | 5 | No |
| 9 | Female | 47 | No | 27 | 26 | 57 | | 20 | 3 | No |
| 10 | Female | 37 | No | 41 | 17 | 83 | | 12 | 3 | No |
| 11 | Male | 42 | Yes | 216 | 157 | 160 | | 35 | 19 | INH+RIF |
| 12 | Female | 74 | Yes | 163 | 127 | 158 | | 57 | 14 | INH+RIF |
| 13 | Male | 61 | Yes | 112 | 98 | 166 | | 43 | 13 | INH+RIF |
| 14 | Male | 65 | Yes | 310 | 317 | 100 | | 33 | 13 | INH+RIF |
| 15 | Male | 67 | Yes | 169 | 150 | 71 | | 52 | 13 | INH+RIF |
| 16 | Male | 55 | Yes | 186 | 111 | 146 | | 61 | 20 | INH+RIF |
| 17 | Male | 77 | Yes | 900 | 380 | 120 | | 87 | 49 | INH+RIF |
| 18 | Male | 65 | Yes | 399 | 358 | 147 | | 31 | 12 | INH+RIF |
| 19 | Male | 78 | Yes | 150 | 190 | 104 | | 42 | 14 | INH+RIF |
| 20 | Male | 72 | Yes | 218 | 337 | 146 | | 57 | 21 | INH+RIF |
| 21 | Male | 53 | Yes | 223 | 192 | 138 | | 52 | 13 | INH+RIF |

**Supplemental Table S3. Primers of genes used for RT-PCR**

| **Mouse** | **Forward (5’-3’)** | **Reverse (5’-3’)** |
| --- | --- | --- |
| Fgf1 | GGGGAGATCACAACCTTCGC | GTCCCTTGTCCCATCCACG |
| Fgf2 | GCGACCCACACGTCAAACTA | TCCCTTGATAGACACAACTCCTC |
| Fgf3 | TGCGCTACCAAGTACCACC | CACCGCAGTAATCTCCAGGAT |
| Fgf4 | TACCCCGGTATGTTCATGGC | TTACCTTCATGGTAGGCGACA |
| Fgf5 | AACTCCTCGTATTCCTACAATCC | CGGATGGCAAAGTCAATGG |
| Fgf6 | CAGGCTCTCGTCTTCTTAGGC | AATAGCCGCTTTCCCAATTCA |
| Fgf7 | TGGGCACTATATCTCTAGCTTGC | GGGTGCGACAGAACAGTCT |
| Fgf8 | GGAACCCAGCTGACACTCTC | TCTTCTGCCATGGCGTTGAT |
| Fgf9 | ATGGCTCCCTTAGGTGAAGTT | TCCGCCTGAGAATCCCCTTT |
| Fgf10 | TTTGGTGTCTTCGTTCCCTGT | TAGCTCCGCACATGCCTTC |
| Fgf15 | GAAGACGATTGCCATCAAGGA | CGAATCAGCCCGTATATCTTGC |
| Fgf17 | GCGGCAAATCCGTGAATACC | GGCCGTGTAGTTGTTCTCCA |
| Fgf18 | GCCCTGATGTCTGCCAAGTA | CCCTTGGGGTAACGCTTCAT |
| Fgf20 | AGGATCACAGTCTCTTCGGTATC | GTCATTCATCCCAAGGTACAGG |
| Fgf21 | TTCAAATCCTGGGTGTCAAA | CAGCAGCAGTTCTCTGAAGC |
| Fgf22 | GGAGATCCGTTCTGTCCGTG | TCCCGGAACCGACCCAT |
| Fgf23 | ATGCTAGGGACCTGCCTTAGA | AGCCAAGCAATGGGGAAGTG |
| Cyp2e1 | TGTGACTTTGGCCGACCTGTTC | CAACACACACGCGCTTTCCTGC |
| Aco | TGGTATGGTGTCGTACTTGAATGAC | AATTTCTACCAATCTGGCTGCAC |
| Cyp4a10 | CAACTTGCCCATGATCACACA | CATCCTGCAGCTGATCCTTTC |
| Nox2 | GAAAACTCCTTGGGTCAGCACT | ATTTCGACACACTGGCAGCA |
| Gcl-c | GTTATGGCTTTGAGTGCTGCAT | ATCACTCCCCAGCGACAATC |
| Gpx-1 | CCAGGAGAATGGCAAGAATGA | TCTCACCATTCACTTCGCACTT |
| Sod-2 | TCCCAGACCTGCCTTACGACTAT | GGTGGCGTTGAGATTGTTCA |
| G6pdh | CTGGAACCGCATCATCGTGGAG | CCTGATGATCCCAAATTCATCAAAATAG |
| Pcna | TTTGAGGCACGCCTGATCC | GGAGACGTGAGACGAGTCCAT |
| Ki67 | AGCACAAAGAGACGGTCTAAGA | CTCTGCCTCGTGACTGTGTT |
| F4/80 | CTTTGGCTATGGGCTTCCAGTC | GCAAGGAGGACAGAGTTTATCGTG |
| Cyp7a1 | GGGATTGCTGTGGTAGTGAGC | GGTATGGAATCAACCCGTTGTC |
| Cyp8b1 | CTAGGGCCTAAAGGTTCGAGT | GTAGCCGAATAAGCTCAGGAAG |
| Cyp27a1 | CCAGGCACAGGAGAGTACG | GGGCAAGTGCAGCACATAG |
| Cyp7b1 | GGAGCCACGACCCTAGATG | GCCATGCCAAGATAAGGAAGC |
| Ntcp | CAAACCTCAGAAGGACCAAACA | GTAGGAGGATTATTCCCGTTGTG |
| Oatp1 | GTGCATACCTAGCCAAATCACT | CCAGGCCCATAACCACACATC |
| Oatp2 | GGGAACATGCTTCGTGGGATA | GGAGTTATGCGGACACTTCTC |
| Bsep | TCTGACTCAGTGATTCTTCGCA | CCCATAAACATCAGCCAGTTGT |
| Mdr2 | CAGCGAGAAACGGAACAGCA | TCAGAGTATCGGAACAGTGTCA |
| Mrp2 | GTGTGGATTCCCTTGGGCTTT | CACAACGAACACCTGCTTGG |
| TNFα | GGCATGGATCTCAAAGACAACC | AAATCGGCTGACGGTGTGG |
| IL-6 | TAGTCCTTCCTACCCCAATTTCC | TTGGTCCTTAGCCACTCCTTC |
| IL-1β | AAATACCTGTGGCCTTGGGC | CTTGGGATCCACACTCTCCAG |
| Hnf4α | CACGCGGAGGTCAAGCTAC | CCCAGAGATGGGAGAGGTGAT |
| Fxr | GCTTGATGTGCTACAAAAGCTG | CGTGGTGATGGTTGAATGTCC |
| Shp | CAGGTCGTCCGACTATTCTGT | AGGCTACTGTCTTGGCTAGGA |
| Lrh-1 | TGTGTGGCGATAAAGTGTCTG | TCGACAGTAGGGACATCGTTT |
| Lxr | CTCAATGCCTGATGTTTCTCCT | TCCAACCCTATCCCTAAAGCAA |
| G6pc | ACTAAAGCCTCTGAAACCC | AGATTCTGCACCGCAAG |
| Pklr | GCACGACTCAACTTCTCCC | CCAGCACCTGTGAACCCT |
| Fgfr1 | TAATACCACCGACAAGGAAATGG | TGATGGGAGAGTCCGATAGAGT |
| Fgfr2 | CCTCGATGTCGTTGAACGGTC | CAGCATCCATCTCCGTCACA |
| Fgfr3 | TGGATCAGTGAGAATGTGGAGG | CCTATGAAATTGGTGGCTCGAC |
| Fgfr4 | TCCATGACCGTCGTACACAAT | ATTTGACAGTATTCCCGGCAG |
| β-Actin | GTGACGTTGACATCCGTAAAGA | GCCGGACTCATCGTACTCC |
| **Human** | **Forward (5’-3’)** | **Reverse (5’-3’)** |
| Cyp7a1 | GCAATTTGGTGCCAATCCTCT | GCACAACACCTTATGGTATGACA |
| Cyp8b1 | GAAGCGCATGAGGACCAAG | TTGCATATTGCCCAAAGTCTAGT |
| Cyp27a1 | GGTGCTTTACAAGGCCAAGTA | TCCCGGTGCTCCTTCCATAG |
| Cyp7b1 | AAAGGTTGGCTTCCTTATCTTGG | GCAACTGACTGATGCTAAATGCT |
| Ntcp | AAGGACAAGGTGCCCTATAAAGG | TTGAGGACGATCCCTATGGTG |
| Oatp1 | GGAGTTGGAACACTGCTCATT | CTTGACTCTAGGAGACACGGA |
| Oatp2 | TTGGAGGTGTTTTGACTGCTT | ACAAGTGGATAAGGTCGATGTTG |
| Bsep | TTGGCTGATGTTTGTGGGAAG | CCAAAAATGAGTAGCACGCCT |
| Mdr2 | CTTGAGGCGGCAAAGAACG | GCCAATCGGAGTATCGAAACA |
| Mrp2 | CCCTGCTGTTCGATATACCAATC | TCGAGAGAATCCAGAATAGGGAC |
| Hnf4α | CACGGGCAAACACTACGGT | TTGACCTTCGAGTGCTGATCC |
| G6pc | GTGTCCGTGATCGCAGACC | GACGAGGTTGAGCCAGTCTC |
| Pklr | TCAAGGCCGGGATGAACATTG | CTGAGTGGGGAACCTGCAAAG |
| β-Actin | CATGTACGTTGCTATCCAGGC | CTCCTTAATGTCACGCACGAT |
